# Mechanistic insights into the multitarget synergistic efficacy of farrerol and β-lactam antibiotics in combating methicillin-resistant *Staphylococcus aureus*

**DOI:** 10.1128/aac.01551-24

**Published:** 2025-02-28

**Authors:** Hangqian Yu, Li Wang, Xin Liu, Jianze Zheng, Hua Xiang, Yanyang Zheng, Dongmei Lv, Jingjing Yang, Yuxin Zhang, Jiazhang Qiu, Dacheng Wang

**Affiliations:** 1 College of Animal Science, Jilin University 12510 , Changchun, China; 2 State Key Laboratory for Diagnosis and Treatment of Severe Zoonotic Infectious Diseases, Key Laboratory for Zoonosis Research of the Ministry of Education, College of Veterinary Medicine, Jilin University 623721 , Changchun, China; 3 Clinical Medical College, Changchun University of Chinese Medicine 159345 , Changchun, China; 4 College of Animal Medicine, Jilin Agricultural University 85112 , Changchun, China; The Peter Doherty Institute for Infection and Immunity, Melbourne, Australia

**Keywords:** methicillin-resistant, *Staphylococcus aureus*, farrerol, penicillin-binding protein 2a, BlaZ, Agr

## Abstract

Methicillin-resistant *Staphylococcus aureus* (MRSA), a principal causative agent of infections worldwide, urgently requires innovative interventions to counter its increasing risk. The present study revealed the profound impact of farrerol (FA), a robust bioactive agent, on the virulence and resistance mechanisms of MRSA. Our in-depth investigation revealed that FA significantly mitigated the β-lactam resistance of MRSA USA300, an achievement attributed to its precise interference with the BlaZ and Pbp2a protein. Additionally, FA indirectly diminishes the oligomerization of PBP2a by disrupting pigment synthesis, further contributing to its efficacy. In addition, FA extends its functional footprint beyond resistance modulation, exhibiting substantial antivirulence efficacy through selective inhibition of the accessory gene regulator (Agr) system, thereby significantly curbing MRSA pathogenicity in A549 cell and murine models. This study comprehensively explored the multiple impacts of FA on MRSA, shedding light on its versatile role as a BlaZ suppressor, pigment synthesis regulator, and AgrA activity modulator. These intricate findings firmly position FA as a compelling therapeutic candidate for addressing MRSA infections in the clinic.

## INTRODUCTION

*Staphylococcus aureus* (*S. aureus*), especially methicillin-resistant *S. aureus* (MRSA), is a principal pathogen in global infections, both within healthcare settings and the community at large (1). Of particular concern is the observed resistance of clinical MRSA isolates to frontline antibiotics, including vancomycin, linezolid, and daptomycin, further restricting therapeutic options (2, 3). The rapid development of drug resistance severely limits the use of β-lactam antibiotics, especially for *S. aureus*, which acquire the *SCCmec* cassette encoding penicillin-binding protein 2a (PBP2a), a protein that has low affinity for β-lactam antibiotics through horizontal gene transfer (4). To bridge this gap, various antimicrobial strategies have been explored, including the use of β-lactamase inhibitors (5, 6), FtsZ inhibitors (7–9), wall teichoic acid (WTA) inhibitors (10–12), MurJ flippase inhibitors (13, 14), and host-acting antibacterial compounds (HACs) (15), which increase bacterial sensitivity to drugs or interfere with pathogen-associated molecular patterns (PAMPs).

It seems more prudent to dismantle the foundation that enables antibiotic-resistant bacteria to become “perfect organisms” rather than permitting their evolution into “perfectly defended bacteria.” To bring β-lactam antibiotics back to the frontline of the battle against *S. aureus* and fill the gap in antibacterial drug development, antimicrobial adjuvants work by suppressing bacterial resistance to existing antibiotics, effectively increasing bacterial sensitivity to other drugs and thereby restoring their clinical utility. A study demonstrates that the triple β-lactam combination of meropenem/piperacillin/tazobactam (ME/PI/TZ) exhibits synergistic and bactericidal activity against MRSA both *in vitro* and *in vivo*. ME/PI/TZ suppresses resistance development through collateral sensitivity and disrupts PBP2a function via an allosteric mechanism. Its comparable efficacy to linezolid highlights the potential of older β-lactam combinations as effective treatments for multidrug-resistant MRSA infections (6). Interestingly, several β-lactamase inhibitors have shown synergistic effects in clinical settings, while therapeutic agents targeting PBP2a activity also exhibit considerable promise. Statin-treated patients have significantly improved outcomes following severe infections (including sepsis, pneumonia, and bacteremia) (16). In *in vitro* and mouse infection models, the use of statins to inhibit staphyloxanthin synthesis resulted in a decrease in PBP2a oligomers on the membrane, enhancing MRSA sensitivity to β-lactam antibiotics (17, 18). Therefore, the use of β-lactam antibiotics in combination with molecules that increase their activity to restore MRSA sensitivity to β-lactam drugs is another feasible strategy. Indeed, the quintessential difficulty in addressing acute infections precipitated by MRSA is not merely the elimination of the pathogenic microorganism but rather the expeditious containment of bacterial proliferation and the mitigation of consequent inflammatory injury within the tissues post-infection (19). The accessory gene regulator (Agr) system orchestrates a pivotal regulatory function in acute *S. aureus* infections and in the modulation of a suite of pore-forming toxins (20). Specifically, the repertoire of toxins produced by Agr governance, including alpha-hemolysin, phenol-soluble modulins, and Panton-Valentine leukocidin (PVL), has been empirically shown to exert a critical influence on the pathogenesis of MRSA-driven afflictions, such as pneumonia, endocarditis, and dermal as well as soft tissue infections (21–23). Despite the documented *in vitro* characterization of numerous quorum sensing inhibitors, the paucity of compounds that exhibit advantageous pharmacological attributes *in vivo* remains unclear (24). Hence, strategic inhibition of the Agr system could attenuate the deleterious impact of host defensive mechanisms attributed to pore-forming toxins, potentially safeguarding the integrity of the indigenous microbiome and enhancing the therapeutic efficacy of conventional antibiotics against invasive systemic infections.

**Fig 1:**
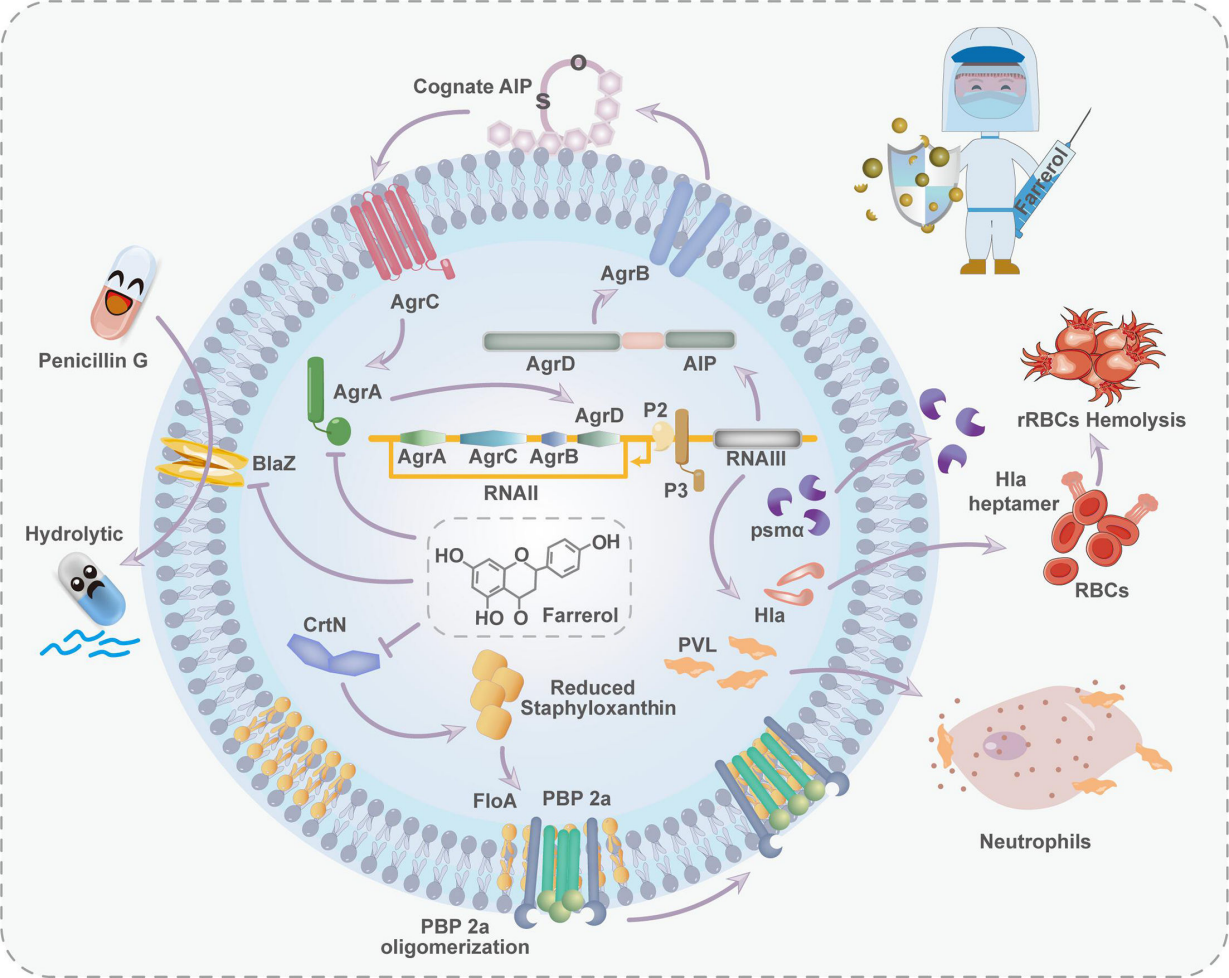
The role of FA in counteracting the resistance and virulence of *S. aureus*. FA significantly enhances the antimicrobial activity of β-lactam antibiotics against MRSA through multiple mechanisms, overcoming its resistance barriers and restoring the efficacy of β-lactams. First, FA inhibits the activity of BlaZ, protecting β-lactam antibiotics from enzymatic degradation and thereby preserving their structural integrity and bactericidal effect. Second, FA disrupts pigment production, further impairing MRSA’s defense mechanisms, and destabilizes the function and oligomerization of PBP2a, a key mediator of cell wall resistance in MRSA. This weakening of PBP2a’s function diminishes its ability to confer resistance to β-lactams. While β-lactams primarily target conventional PBPs and have limited affinity for PBP2a, FA enhances the ability of β-lactams to inhibit MRSA cell wall synthesis by destabilizing and impairing PBP2a. Additionally, FA modulates the expression of virulence genes, including those regulated by the Agr system, leading to reduced production of virulence factors such as Hla and PVL. This suppression of virulence decreases MRSA’s adhesion, invasion, and immune evasion capabilities, thereby further weakening its pathogenicity. Through these synergistic actions, FA not only restores MRSA’s sensitivity to β-lactams but also exemplifies the potential of natural compounds to overcome antibiotic resistance, offering a powerful tool in the fight against multidrug-resistant and virulent bacterial pathogens.

In this study, we pioneered a comprehensive exploration of the putative molecular mechanism underlying the action of farrerol (FA) on *S. aureus*. Our findings affirm that FA can effectively curtail β-lactamase activity, attenuate PBP2a oligomerization by hindering pigment production, and act as a potent inhibitor of the *S. aureus* agr system. Importantly, FA is a dual-action inhibitor that can concurrently diminish virulence and reverse antibiotic resistance (Fig. 1). These findings establish a formidable theoretical basis for the future refinement and clinical application of FA, thereby creating a new strategy for anti-MRSA therapy.

## MATERIALS AND METHODS

### Strains and reagents

The bacterial strains involved in this study are detailed in Table S1. *S. aureus* strains were grown in TSB or on tryptic soy agar (TSA) plates without glucose supplementation. *Escherichia coli* strains were grown in Luria Bertani (LB) broth or on LB agar plates. Unless otherwise noted, the cultures were incubated at 37°C in a shaker at 250 rpm for aeration. Cefepime, cefoxitin, ceftiofur, cefotaxime, latamoxef, dicloxacillin, penicillin G, and ampicillin were obtained from the State Food and Drug Administration of China. FA (purity >98%) was purchased from RuiFensi Biotech Co., Ltd. (Chengdu, China).

### Minimum inhibitory concentration (MIC)

The MICs of FA or antibiotics against *S. aureus* USA300 were measured using the broth microdilution method (25). All the experiments were performed in 96-well plates with 100 µL of reaction mixture containing different concentrations of the drug. *S. aureus* (1 × 10^5^ CFU/mL) was subsequently added to each well. Moreover, culture medium alone was used for the control group. The plates were incubated for 16 h, after which the OD_600_ was determined via a microplate reader.

### Checkerboard microdilution assay

The antibacterial synergy between FA and antibiotics was evaluated using a checkerboard assay as described previously (26). In detail, 100 µL of FA or antibiotics was serially diluted twofold. Then, 10^5^ CFU/mL *S*. *aureus* was added to each well, and the plates were incubated for 16 h. The potential synergistic effect was determined according to the fractional inhibitory concentration index (FICI), which was calculated using the following equation:


$$\frac{MIC\;Farrerol\;combination}{MIC\;Farrerol\;alone}+\frac{MIC\;antibiotic\;combination}{MIC\;antibiotic\;alone}$$

The FIC index values were interpreted as follows: FICI ≤0.5 indicates synergism, 0.5 < FICI ≤ 4.0 indicates additive, and FICI >4 indicates antagonism. On the basis of the FICIs, the best antibiotic was chosen for the following experiments (27).

### *In vitro* time‒kill curves

To further investigate the bactericidal activity of the antimicrobial drugs, time‒kill kinetic curve assays were performed with different exposure durations (28). Briefly, USA300 (1 × 10^6^ CFU/mL) was exposed to FA or cefepime alone or in combination and incubated at 37°C for six different periods (0, 2, 4, 8, 15, and 24 h). Colony counts were performed on solid plates diluted at appropriate dilutions.

### LDH release assay

Calcein AM is a fluorescent dye that is hydrolyzed by intracellular esterases in viable cells, resulting in a green fluorescent signal, which specifically stains live cells. Propidium iodide (PI), in contrast, is a red fluorescent dye that binds to DNA but is impermeable to the membranes of live cells, making it useful for identifying dead cells. The LDH release assay was conducted according to methods previously described in the literature (29). A549 cells were cultured in 1640 medium supplemented with 10% fetal bovine serum (FBS) at 37°C in a 5% CO_2_ atmosphere. The cells were seeded at a density of 5 × 10^4^ cells per well in 24-well plates and allowed to grow for 24 h. Overnight cultures of *S. aureus* USA300 were diluted, grown to an OD_600_ of 0.5, centrifuged, and then resuspended in 1640 medium without FBS or antibiotics. The cells were cocultured with *S. aureus* USA300 and FA at concentrations ranging from 0 to 64 µg/mL, followed by incubation at 37°C for 3 h. The supernatants were then collected for analysis. LDH release was determined using an LDH assay kit (Beyotime, Shanghai, China).

### Calcein/PI fluorescence staining

A549 cells were cultured in 1640 medium containing 10% FBS at 37°C with 5% CO_2_. The cells were seeded at a density of 5 × 10^4^ cells per well in 24-well plates and cultured for 24 h. Overnight cultures of *S. aureus* USA300 were diluted, cultured to an OD_600_ of 0.5, centrifuged, and resuspended in 1640 medium (without FBS/antibiotics). The cells were cocultured with *S. aureus* USA300 and FA, incubated (37°C, 3 h), stained using calcein AM/PI kit (Beyotime, Shanghai, China), and subsequently washed with phosphate-buffered saline (PBS) (29). Calcein AM is a cell-permeable dye that is converted into green-fluorescent calcein by intracellular esterases in live cells, indicating cell viability. PI is a red fluorescent dye that only penetrates cells with compromised membranes, marking dead cells. Taken together, these dyes allow for the distinction between live (calcein-positive) and dead (PI-positive) cells during viability assessments using fluorescence microscopy. Cell viability was determined via inverted fluorescence microscopy.

### Hemolytic effects on rabbit and goat blood cells

Different concentrations of FA were assessed by mixing 25 µL of either rabbit or defibrinated goat blood with 975 µL of PBS. Triton X-100 was used as the hemolysis standard. Triton X-100 is a widely employed nonionic surfactant due to its potent ability to disrupt and solubilize cell membranes, making it a reliable positive control for inducing hemolysis. PBS was used as a nonhemolytic control. Following incubation at 37°C for 1 h and centrifugation, the absorbance of the supernatant at 543 nm was measured to analyze the hemolytic potential of FA (30).

### Toxicological evaluation in *Galleria mellonella* models

Mature larvae of *G. mellonella*, with a mass range of 220–260 mg, were reared under stringent temperature control at 37°C (31). These larvae were then evenly distributed into four distinct groups, each comprising 10 larvae: one group received no treatment, which served as the baseline control; the other was exposed to the solvent as a control; and the final two groups were administered FA at concentrations of 25 and 50 mg/kg. The baseline control group underwent no procedural treatments. Administration was carried out using the use of a 10 µL Hamilton syringe to inject either PBS mixed with 0.1% DMSO or the specified concentrations of the drug into the terminal proleg of each larva. Over the following 5 days, observations were made regarding the survival rates and melanization responses of the larvae across all groups, and the results were meticulously recorded.

### Cloning, protein expression, and purification

Using polymerase chain reaction (PCR), a DNA fragment encoding *hla* was amplified from the genomic DNA of the *S. aureus* USA300 strain. The primers used for this purpose, *hla*-clone-F and *hla*-clone-R, included *bamHI* and *xhoI* sites (underlined). The amplified product was subsequently inserted into the *bamHI* and *xhoI* sites of the pET28a vector. Similar construction methodologies were employed for *agrA*_C_, *agrC_C_*, and *blaZ*, and the primers used are detailed in Table S2.

The expression and purification processes for the recombinant Hla, BlaZ, AgrA_C_, and AgrC_C_ proteins were identical to the methods described below. To express the respective proteins, *E. coli* BL21(DE3) carrying the expression plasmids were cultured in LB media at 37°C until the optical density at 600 nm reached 0.8–1.0. After the culture was chilled on ice for 30 min, isopropyl β-d-1-thiogalactopyranoside (IPTG) was added to a final concentration of 0.25 mM to induce protein expression. The cultures were then incubated at 18°C for 24 h, followed by centrifugation at 6,000 × *g* for 10 min at 4°C. The collected bacterial pellets were resuspended in buffer A containing 1 mM phenylmethylsulfonyl fluoride (20 mM Tris-HCl pH 8.0, 150 mM NaCl, and 10 mM imidazole) and lysed via a high-pressure cell disruptor at low temperature. The lysate was subsequently centrifuged at 12,000 × *g* for 20 min at 4°C for visualization. The supernatant was purified via a HisTrap HP column and washed with 50 mL of buffer A containing 20 mM imidazole, after which the target proteins were eluted with 3 mL of buffer A containing 0.25 M imidazole. The eluates containing the target proteins were dialyzed in storage buffer (25 mM Tris-HCl [pH 8.0] and 100 mM NaCl) at 4°C for 4 h, concentrated to 10 mg/mL, and stored at −80°C until further use.

### Hydroxylamine assay

Penicillinase activity was tested via a hydroxylamine assay as previously described (32). Penicillin G was dissolved in sodium citrate and HCl buffer. Various volumes (50, 100, 200, 250, 500, and 750 µL) of these solutions were subsequently added to the HCl buffer to a final volume of 1 mL. Then, 1 mL of neutral light amine and 1 mL of 95% ethanol were added, followed by the addition of 2 mL of ferric ammonium sulfate after a 3-min reaction. The OD_515_ was measured once the bubbles disappeared, and buffer was used as a blank control. The crude enzyme mixture was mixed with FA (0–128 µg/mL) and penicillin G solution (2,000 μg/mL) and incubated at 37°C for 30 min, after which the OD was measured. The penicillin G concentration was calculated on the basis of the standard curve. Clavulanic acid (0–100 µg/mL) served as a positive control.

### Transmission electron microscopy (TEM)

TEM was used to visually assess the morphological changes in MRSA after treatment (33). When *S. aureus* USA300 was cultured to an OD_600_ of 0.2, 1/8× MIC FA was added to the *S. aureus* culture, and the samples were cultured for 4 h. Meanwhile, untreated *S. aureus* served as a control group. The bacteria were subsequently collected via centrifugation (5,000 × *g*, 5 min) and washed with PBS. Subsequently, 4 mL of 2.5% (vol/vol) glutaraldehyde was added, and the samples were incubated overnight at 4°C for immobilization of the bacteria. Ultrathin sections were stained and observed with a Hitachi H-7650 transmission electron microscope.

### WTA synthesis

MRSA USA300 bacterial suspensions treated with different concentrations of FA (8, 16, 32, and 64 µg/mL) were collected, washed with Buffer 1, and centrifuged. The samples were then boiled for 1 h, and the bacterial remnants were recovered via centrifugation. Each sample was then sequentially washed with Buffers 2, 3, and 1. After treatment with proteinase K, the cells were incubated at 50°C for 4 h, resuspended in 0.1 M NaOH, oscillated at room temperature for 16 h, and centrifuged for PAGE analysis. Gel staining was performed using the silver staining method (34).

### Staphyloxanthin biosynthesis inhibition assay

Overnightly *S. aureus* cultures were diluted 1:100 in TSB culture medium, and FA (0–64 µg/mL) was added to each 2 mL tube at a tube-to-medium volume ratio of 10:1. After incubation for 24 h, 1.5 mL of the bacterial culture was collected and centrifuged. An equal amount of DMSO was added as a control, followed by visual analysis of the pigmentation of *S. aureus* (35).

### Measurement of pigment production

*S. aureus* was incubated in TSB (4 mL) supplemented with FA (0–64 µg/mL) for 48 h in a shaker at 250 rpm. Subsequently, 3 mL of the bacterial culture was centrifuged and washed twice with 0.01 M PBS. The pigment was extracted three times with methanol, and methanol was added to a total volume of 1 mL. The absorbance was measured at 450 nm via a UV spectrophotometer (35).

### Hydrogen peroxide killing assay

*S. aureus* USA300 was cultured overnight and inoculated at a 1:100 ratio in fresh BHI, followed by a 24-h incubation with or without 64 µg/mL FA. After incubation, the bacterial suspension was centrifuged, washed, and diluted to 1 × 10^7^ CFU/mL. Hydrogen peroxide was added to a final concentration of 1.5%, and the samples were incubated for 1 h. The reaction was terminated by the addition of catalase (1,000 U/mL), and bacterial viability was assessed by CFU counts on a TSA plate (36).

### Absorbance analysis of staphyloxanthin

Methanol extracts containing staphyloxanthin from both the untreated control group and MRSA treated with FA (64 µg/mL) were prepared. The absorbance spectra of these staphyloxanthin extracts were meticulously recorded across a comprehensive wavelength range of 350–650 nm via a spectrophotometer (Thermo, USA) (35).

### Spectrometric quantification of staphyloxanthin biosynthesis intermediates

The quantification of staphyloxanthin intermediates in the collected supernatant was achieved via absorption spectroscopy at distinct wavelengths via a multilabel reader (Thermo, USA). The absorption measurements were conducted at specific wavelengths: 286 nm for 4,4′-diapophytoene, 435 nm for 4,4′-diaponeurosporene, 455 nm for 4,4′-diaponeurosporenic acid, and 462 nm for staphyloxanthin (37). Naftifine was used as the control for these measurements (38).

### Oligomerization of PBP2a

USA300 was cultured overnight, diluted 1:100 in fresh BHI, and grown with or without ibandronate (100 µM) or FA (64 µg/mL). Ibandronate inhibits IspA, blocking FPP synthesis and disrupting pigment production, thereby interfering with PBP2a oligomerization, and was used as a positive control in this study (17, 39). After being harvested, the bacteria were washed and resuspended in PBS supplemented with 1 mM PMSF and complete protease inhibitor. The membrane protein (~30 mg) was extracted from 50 mL of the bacterial suspension. The samples were cross-linked, freeze‒thawed, treated with lysostaphin, and disrupted. After centrifugation, the supernatant was ultracentrifuged for membrane isolation and protein quantification. The membrane fractions were solubilized with sample buffer containing 0.5% DDM, mixed with Coomassie Blue G-250, and subjected to gel electrophoresis. Western blotting analysis was used to determine PBP2a protein levels.

### *S. aureus* hemolysis assay

Overnight-cultured *S. aureus* USA300 was diluted 1:100 until the OD_600_ reached 0.3, treated with 64 µg/mL FA and incubated at 37°C and 220 rpm until the OD_600_ reached 2.5. The culture supernatant was harvested (5 min, 5,000 rpm) and mixed with washed rabbit blood in EP tubes. The controls included PBS or a mixture of Triton X-100 and PBS. After incubation at 37°C for 1 h, the samples were centrifuged (5 min, 4,000 rpm), and the supernatant absorbance was measured at 543 nm in a 96-well plate via an MD SPECTRA Max reader (40).

### Western blotting analysis

*S. aureus* was cultured in the presence of different concentrations of FA until late logarithmic growth was reached, and the supernatant of each group of cultures was collected. The proteins were subsequently transferred to PVDF membranes after conventional SDS‒PAGE. Then, the PVDF membrane was blocked at room temperature for 2 h. Immediately thereafter, a 1:3,000 dilution of rabbit anti-Hla antibody was added, and the membrane was incubated on a shaking table at room temperature for 2 h. This was followed by three 10-min washes with PBST. A 1:5,000 dilution of HRP-labeled sheep anti-rabbit secondary antibody was then added, and the membrane was incubated at room temperature for 2 h. After the secondary antibody was removed, the membrane was washed three times. Finally, an enhanced chemiluminescence color development solution was added, after which the membrane was exposed and imaged under a developer (41).

### Hla oligomerization assay

Purified recombinant Hla protein was treated with 5 mM sodium deoxycholate and various FA concentrations (0–64 µg/mL) for 25 min at 22°C. Next, 5× protein loading buffer, excluding β-mercaptoethanol, was added to the mixture, which was then incubated at 55°C for 10 min. The samples were subjected to analysis via 10% SDS–PAGE, and the gel was subsequently stained with Coomassie Brilliant Blue. After decolorization, the gel was placed under a gel imager (Bio-Rad) to observe heptamer formation (40).

### *In vitro* phosphorylation assay

The *in vitro* phosphorylation assay was conducted as previously described (42, 43). To maintain a constant concentration of 10% DMSO across all phosphorylation reactions, a series of dilutions was prepared by diluting FA at 10 mg/mL in 100% DMSO. In a standard autophosphorylation reaction with a volume of 10 µL, purified AgrC_C_ protein (5 µM) was incubated with 1 µL of the diluted drug or 100% DMSO in reaction buffer (50 mM Tris-HCl pH 8.0, 50 mM KCl, 0.5 mM DTT, and 5 mM MgCl_2_) at 30°C for 15 min. The reaction was initiated by adding ATPγS to a final concentration of 130 µM, followed by further incubation at 30°C for 45 min. Subsequently, p-nitrophenyl mesylate was added to achieve a final concentration of 1 mM, facilitating the formation of thiophosphate ester sites, and the mixture was incubated at room temperature for 2 h. The reaction was halted by the addition of loading buffer. For SDS‒PAGE analysis, approximately 0.5 µg of protein was loaded into each well of a 12.5% SDS‒polyacrylamide gel. Western blot analysis was performed using an anti-thiophosphate ester antibody to determine the phosphorylation status of AgrC_C_.

### RT‒qPCR

*S. aureus* USA300 was cultured in BHI supplemented with DMSO or various FA concentrations and incubated (37°C, 180 rpm, OD_600_ exceeded 2.0). Total RNA was extracted using TRIzol and reverse transcribed to cDNA using the BeyoRT II Kit. The transcript levels of *crtN*, *agrA*, *RNAIII*, and Hla were analyzed using qPCR with NovoStart SYBR qPCR SuperMix Plus, normalized to *16S RNA*, and calculated using the 2^−ΔΔCt^ method (44). The primers used are listed in Table S2.

### Molecular electrostatic potential analysis

The study commenced with the minimization of the molecular structure via Chem3D’s MMFF94, aiming to find the global energy minimum through torsional angle adjustments. This optimized structure was subsequently analyzed in Gaussian, where geometric optimizations were conducted at the B3LYP/6-31G(d,p) level. Concurrently, conformational analyses to explore energetically favorable structures were performed with Gaussian 09 W, optimizing each conformer in vacuum at the same theoretical level. This streamlined approach merges molecular mechanics with quantum chemistry to elucidate the electrostatic characteristics of molecules, providing insights into their intermolecular interactions.

### Molecular docking

Molecular simulations were performed on the primary amino acid sites involved in the binding of FA to BlaZ and AgrA. The crystal structures of the BlaZ (PDB ID: 1ALQ) and AgrA (PDB ID: 3BS1) (44) proteins were obtained from the Protein Data Bank. The 3D structure of FA was rendered using ChemBioDraw Ultra. AutoDockTools was used for docking preparation, including nonpolar hydrogen atom merging and rotatable bond specification in the ligand structures (45).

### Intracellular thermal shift assays (CETSAs)

BL21-pET28a::*agrA_C_* was cultured and lysed after the induction of AgrA_C_ protein expression. Total protein was isolated from the supernatant and treated with 64 µg/mL FA or DMSO (37°C, 1 h). The resulting supernatant was equally split into six 60 µL aliquots, each subjected to a specific temperature for 5 min (25°C, 35°C, 39.7°C, 43.4°C, 43.7°C, and 50°C). After incubation, the samples were centrifuged, the supernatant was collected, and the AgrA_C_ protein content at each temperature was analyzed via SDS‒PAGE and Coomassie brilliant blue staining (46).

### Thermal shift assays (TSAs)

TSAs were generated in PCR tubes by combining AgrA_C_ protein (final concentration of 0.04 mg/mL), 100× SYPRO orange, FA (64 µg/mL), and TSA buffer to a total volume of 50 µL. A real-time PCR detection system was used, and the sample was heated from 25°C to 90°C at a rate of 1°C per min. The fluorescence intensity of the proteins as a function of temperature change was recorded (46).

### Fluorescence quenching

Recombinant protein (500 ng/mL in PBS) and various concentrations of a small molecule inhibitor were mixed in quartz cuvettes. Following a 1-min incubation at room temperature, fluorescence spectra were measured (excitation at 280 nm, recording from 280 to 400 nm). The Stern–Volmer constants were calculated from the intensity ratios (*F*_0_/*F*) according to the standard equation. Linear regression of *F*_0_/*F* against the inhibitor concentration provided the binding affinity constant (46).

### Electrophoretic mobility shift assays (EMSAs)

The double-stranded P3 promoter sequence was synthesized by annealing at 95°C for 3 min. EMSA was performed using purified AgrA_C_, FA, and a P3 DNA probe labeled with 3′6-fluorescein. AgrA_C_ (at a concentration of 20 µM) was incubated at 25°C for 20 min in reaction buffer supplemented with either DMSO or varying concentrations of FA. Subsequently, 1 µL of P3 DNA duplex was added to each sample, and the mixture was incubated for an additional 20 min. The samples were then subjected to 8% native PAGE in TBE buffer under dark conditions to prevent photobleaching (46).

### Mouse pneumonia model

To determine the therapeutic effect of the combination of FA and cefepime on pneumonia in mice (47), C57BL/6J female mice (7 weeks), weighing approximately 20–25 g (*n* = 8), were provided by the Experimental Animal Center of Jilin University. A mouse model of *S. aureus* pneumonia was established as previously described. The amount of bacteria administered to the mice was determined according to previous methods. Briefly, anesthesia was induced with ether, and the mice were inoculated with a suspension of *S. aureus*, which was delivered to infected lung tissue via the nasal route. To evaluate the survival rate of the mice, the mice were inoculated with 30 µL (2 × 10^8^ CFUs) of bacterial suspension (47). At 1 h after inoculation, FA (50 mg/kg) was intraperitoneally injected, and cefepime (50 mg/kg) or the combination was subcutaneously injected every 12 h; the 96 h survival rate was subsequently evaluated. For histopathological analysis and bacterial load experiments, the mice were infected with 30 µL of *S. aureus* (1 × 10^8^ CFU). After inoculation, FA (50 mg/kg), intramuscular cefepime (50 mg/kg), or the combination of the two agents was injected subcutaneously every 12 h. After 24 h, lung tissue was excised from all anesthetized mice under aseptic conditions. The right lung tissue was homogenized for colony formation unit (CFU) assessment via serial dilution, which is essential for bacterial quantification. Simultaneously, the left lung was fixed in 10% formalin overnight and stained with hematoxylin and eosin (H&E) or LY6G to evaluate the tissue structure and neutrophil levels, respectively, to aid in histopathological analysis. Furthermore, the remaining left lung lobes were analyzed for wet‒dry weight ratios to assess fluid accumulation.

### Statistical analysis

Each test was repeated three times, and the data are expressed as the means ± SDs, with *P* < 0.05 considered to indicate statistical significance. GraphPad Prism 8.0 (GraphPad Software, Inc., San Diego, USA) was used to analyze the data. Two-sample comparisons were performed via Student’s *t* test (normal distribution) or the Mann‒Whitney *U* test (abnormal distribution). For multiple group sample comparisons, one-way ANOVA or nonparametric tests were used, whereas for pairwise comparisons, Dunnett’s multiple comparisons test was used. Survival curves were analyzed using the log-rank test.

## RESULTS

### FA exhibits synergistic anti-MRSA activity with β-lactam antibiotics and enhances cell survival as a monotherapy in MRSA infections

**Fig 2:**
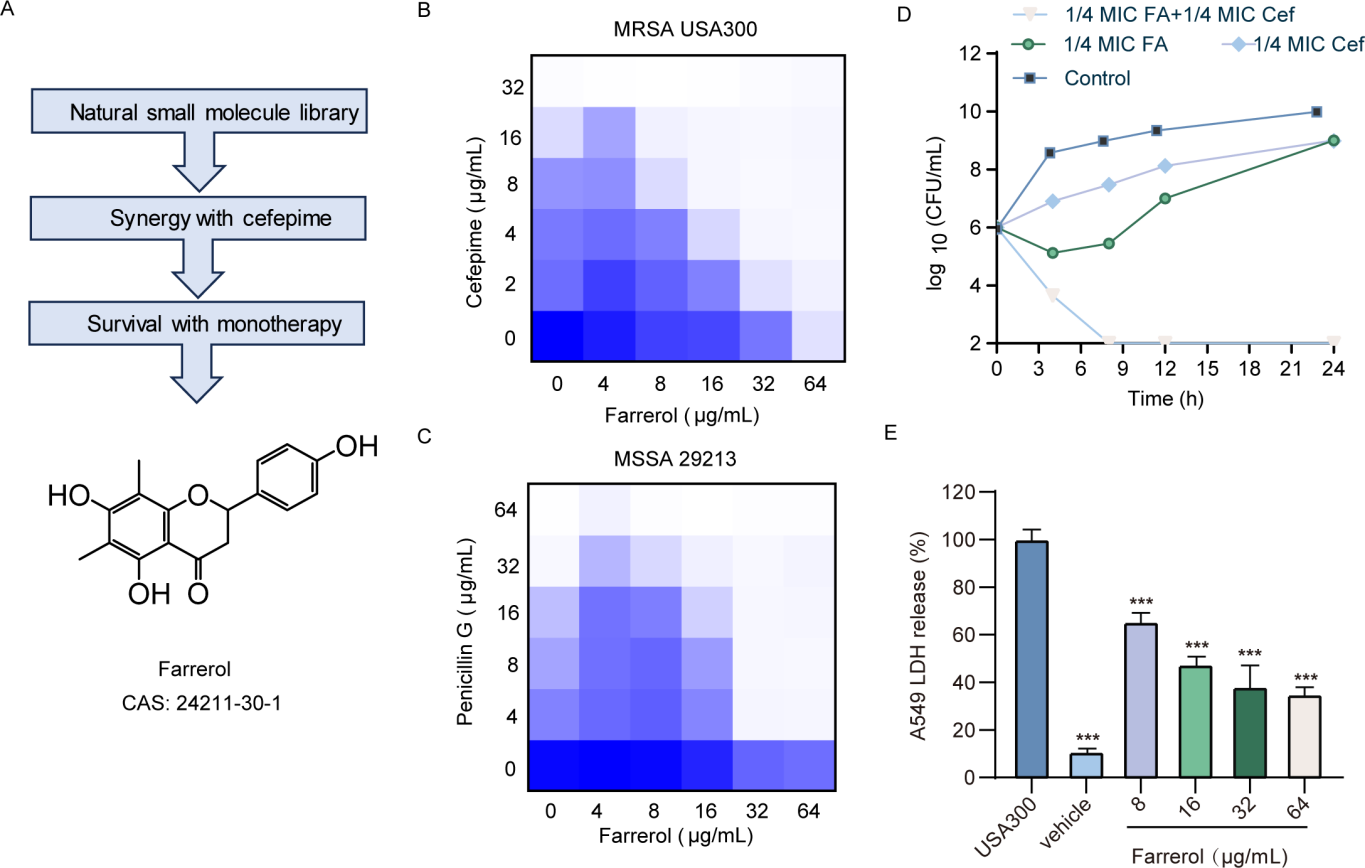
FA exhibits synergistic anti-MRSA activity with β-lactam antibiotics and enhances cell survival as a monotherapy for MRSA infections. (**A**) The workflow implemented in this study identified FA as a candidate drug. (**B**) A representative checkerboard assay demonstrating the interaction between cefepime (1/4 MIC) and FA (1/8 MIC) against *S. aureus* USA300 (FICI = 0.375). (**C**) A representative checkerboard assay depicting the synergistic effect of penicillin G (1/8 MIC) and FA (1/8 MIC) on *S. aureus* 29213 (FICI = 0.25). Each checkerboard assay was performed independently three times, all of which produced consistent results. (**D**) Time‒kill kinetics for USA300 subjected to various concentrations of cefepime and FA in combination (1/4 MIC of cefepime and 1/4 MIC of FA) demonstrated significant bactericidal activity. (**E**) LDH analysis was conducted to evaluate the effects of various concentrations of FA on the survival of A549 cells infected with MRSA USA300. *** represents *P* < 0.001.

We initiated a screening process using a library of small molecules derived from various plants to identify synergistic effects of cefepime at a concentration of 8 µg/mL, aiming for a fourfold reduction from the MIC of 32 µg/mL against the MRSA strain USA300. To minimize the impact of spontaneous mutations, the inoculum density was maintained between 2 × 10^5^ and 5 × 10^5^ CFU. Furthermore, we evaluated the effects of candidate drugs at a concentration of 64 µg/mL on lactate dehydrogenase (LDH) levels in A549 cells infected with *S. aureus* and compared these results to those of a control group. Our findings conclusively demonstrated that FA has a significant synergistic effect on the secondary screening, as illustrated in Fig. 2A. The MIC of cefepime against MRSA USA300 was 32 µg/mL, whereas FA alone had an MIC of 512 µg/mL. When FA (1/8 MIC) was combined with cefepime, the efficacy of cefepime against USA300 was significantly enhanced, resulting in a fourfold reduction in the MIC, thus demonstrating a strong synergistic effect. FA exhibited a notable synergistic interaction with cefepime against MRSA (FICI = 0.375) (Fig. 2B; Table S3). Notably, the checkerboard assay results showed that the MIC of penicillin G against MSSA 29213 was 32 µg/mL. However, in the presence of 1/8 MIC FA, penicillin G at a reduced concentration of 4 µg/mL exhibited synergistic antibacterial effects against MSSA (FICI = 0.25) (Fig. 2C; Table S3). Cefepime, which has a pronounced synergistic effect when combined with FA, was selected for further analysis. Bactericidal testing revealed that total elimination of USA300 was achieved within 8 h of treatment, with a 1/4 MIC of both agents (Fig. 2D). Coadministration of 1/8 MIC cefepime with 1/8 or 1/16 MIC FA led to sustained inhibition of USA300 growth over 24 h (Fig. S2).

*S. aureus* secretes many pore-forming toxins that initiate inflammatory responses and activate inflammatory cells. These activated cells, in response, secrete a variety of cytokines, leading to further inflammatory reactions (48, 49). *S. aureus* exploits inflammasomes to activate pathways leading to host cell death, including pyroptosis, necroptosis, and apoptosis (50, 51). In this study, we evaluated the impact of FA on LDH release and cell viability in A549 cells infected with *S. aureus*. After treatment with 8 µg/mL FA, LDH release decreased to 65.6%, indicating decreased cell damage. As the concentration of FA increased to a synergistic level of 64 µg/mL, a further decrease in LDH release was observed, dropping to 35.1% (Fig. 2E). We evaluated the potential protective effects of FA on USA300 infection in A549 cells via a calcein/PI dual fluorescence staining assay. As anticipated, USA300 elicited considerable cell death in susceptible A549 cells. However, upon treatment with FA, a remarkable reduction in cellular mortality was observed, revealing a pronounced dose-dependent relationship (Fig. S3). These findings suggest that FA mitigates the cytotoxic effects induced by *S. aureus*, potentially through the modulation of inflammatory pathways and a reduction in host cell death.

Notably, while FA has demonstrated some degree of toxicity toward cancer cells in previous studies (52), we did not observe similar toxic effects in rabbit or goat red blood cells, even at concentrations significantly higher than those used in prior research. Additionally, similar outcomes were obtained when the strains were tested in *G. mellonella* (Fig. S4).

### Mode of action determination in synergy

**Fig 3:**
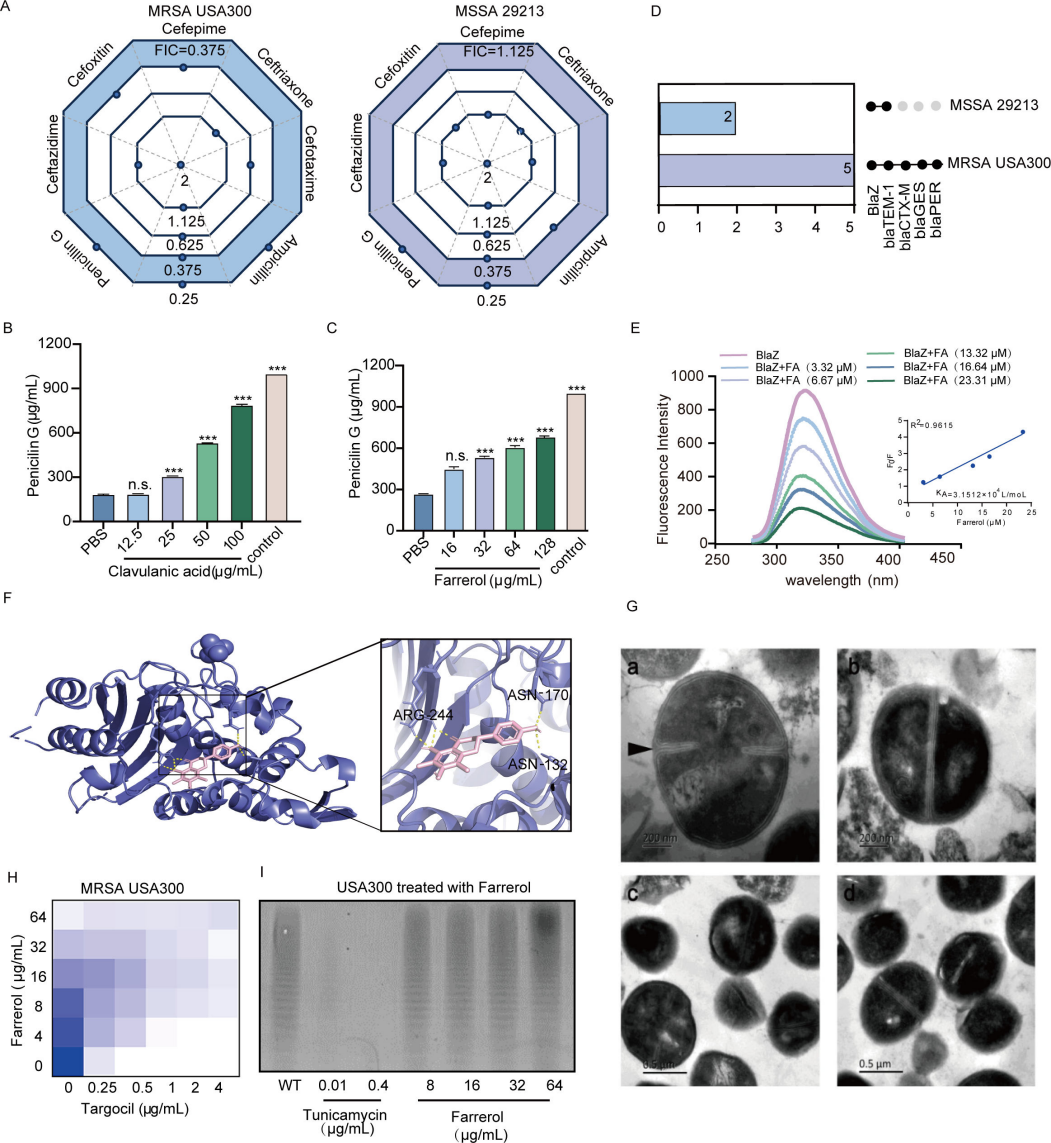
Interaction of FA with the blaZ protein and its lack of effect on WTA formation and cell division. (**A**) Checkerboard analysis was used to assess the combined antimicrobial action of FA and β-lactamase inhibitors against MRSA USA300 and MSSA 29213, as determined by the fractional FICI. (**B, C**) Utilization of the hydroxylamine method to discern the influence of clavulanic acid and FA on β-lactamase activity in the lysate of *S. aureus* USA300. (**D**) Distribution of β-lactamases within *S. aureus* USA300 and 29213. (**E**) Fluorescence quenching assay to determine the binding affinity of FA (0–23.31 μM) for BlaZ. *F*₀*/F* represents the fluorescence quenching ratio. Here, *F*₀ is the initial fluorescence intensity of the protein before the addition of the drug, while *F* is the fluorescence intensity of the protein after drug treatment. *R*² represents the coefficient of determination. In fluorescence quenching experiments, *R*² measures the linear correlation between drug concentration and changes in fluorescence intensity. The value of *R*² ranges from 0 to 1, with values closer to 1 indicating a strong linear relationship between drug concentration and fluorescence intensity. (**F**) A docking model illustrating the interaction between FA and BlaZ. Molecular docking experiments indicated that the potential binding sites of FA are located within the catalytic cavity of BlaZ. (**G**) TEM images of *S. aureus* treated with FA (a, c for the control groups; b, d for the FA-treated groups). The black arrows indicate a continuous, intensely stained, electron-dense layer on the surface, which represents the WTA-rich peptidoglycan (PG) layer. (**H**) Representative checkerboard assay of targocil and FA against *S. aureus* USA300. () Silver-PAGE assay to determine the effects of tunicamycin and FA on the synthesis of WTA in *S. aureus* USA300. n.s. represents no significant difference; *** represents *P* < 0.001.

To further determine the mechanism underlying the synergistic antibacterial activity of FA and β-lactam antibiotics, we evaluated the fractional inhibitory concentration (FIC) index against USA300 upon coadministration of several antibiotics with FA at a concentration of 64 µg/mL. FA, in combination with various antibiotics, displayed synergistic antimicrobial action against MRSA, with FIC indices ranging between 0.25 and 0.375 (Fig. 3A). We subsequently expanded our scope to investigate the potential for similar synergistic effects against methicillin-sensitive *S. aureus* (MSSA) strains. Because of the synergistic effects of FA and MSSA, we first investigated the impact of FA on β-lactamase activity in *S. aureus* using the hydroxylamine method. Penicillin G degradation analysis and β-lactamase activity assessment are shown in Fig. S5. The results of the hydroxylamine method revealed that FA, much like the well-known β-lactamase inhibitor potassium clavulanate, provides a comparable level of β-lactamase inhibition in a concentration-dependent manner in the *S. aureus* lysate (Fig. 3B). These findings underscore the promising potential of FA as an effective β-lactamase suppressor. This finding suggested that FA may target the same variant of β-lactamase in *S. aureus* as potassium clavulanate does, most likely the class A β-lactamase (Fig. 3C,D).

Given that FA can inhibit β-lactamase activity in *S. aureus* lysates, we sought to further assess whether FA exerts its effects via direct interaction with BlaZ, a β-lactamase common to USA300 and 29213. Fluorescence quenching, which is based on the principle of static quenching following small molecule–protein binding, was employed to detect direct interactions. With increasing concentrations of FA (0–23.31 μM), the BlaZ fluorescence gradually decreased, displaying a linear correlation with the FA concentration (*R*^2^ = 0.9615). The calculated *K*_A_ value was 3.1512 × 10^4^ L/mol, indicating that FA strongly interacted with BlaZ (Fig. 3E). Molecular docking simulations suggested that FA binds to three conserved sites within the four conserved sequences of the catalytic cavity of BlaZ, hindering the entry of substrates through the loop structure. Furthermore, FA interactions with the Asn 132, Asn 170, and Arg 244 residues are positioned in three force directions, stabilizing its presence within the BlaZ cavity. The calculated total binding free energy (Δ*G*_bind_) was −8.1 kcal/mol (Fig. 3F). In summary, these results indicate that FA and BlaZ strongly interact. FA likely functions by directly binding to and inhibiting BlaZ, preventing β-lactam antibiotic hydrolysis and leading to a synergistic antibacterial effect.

Notably, the combination of FA with certain antibiotics had synergistic effects only on MRSA, while no synergistic effects were observed for MSSA. This leads us to postulate that FA may target, either directly or indirectly, the pathways related to PBP2a—a protein encoded by the mecA gene, which is exclusive to MRSA. PBP2a regulation is associated with the synthesis of WTA in MRSA, septum formation, and functional membrane microdomain (FMM). Observation of bacterial cell morphology at different stages under treatment with 64 µg/mL FA via TEM was also conducted to assess the effect of FA on bacterial cell wall integrity (Fig. 3G). The cells from the negative control group exhibited a continuous, intensely stained, electron-dense layer on their surface, which was WTA-rich peptidoglycan (PG), and they exhibited symmetric division. However, most cells under FA treatment lacked this complete intensely stained layer, with blurred edges and a reduced WTA layer density. To further determine whether the decrease in WTA layer density caused by FA is linked to WTA synthesis, we utilized targocil, a WTA synthesis inhibitor, as a probe in our experiment. We analyzed the inhibitory effect of the combination of targocil and FA on MRSA via a heatmap. As the concentration of FA increased, the inhibitory effect of targocil on MRSA USA300 diminished, and the MIC gradually increased, suggesting that FA counteracts the inhibitory effect of targocil on MRSA, possibly by acting on an early-stage WTA synthesis inhibitor (Fig. 3H). The impact of FA on the amount of WTA synthesized was subsequently determined through silver-stained PAGE. We used the WTA synthesis inhibitor tunicamycin as the positive control in our study. Our findings indicated that 0.01 µg/mL tunicamycin significantly inhibited WTA synthesis and that 0.4 µg/mL tunicamycin completely suppressed WTA synthesis. However, with increasing FA concentration, there was no observable decrease in WTA synthesis, indicating that FA does not affect WTA synthesis (Fig. 3I). In summary, FA does not affect WTA synthesis or cell division.

### FA interferes with pigment production and PBP2a oligomerization

**Fig 4:**
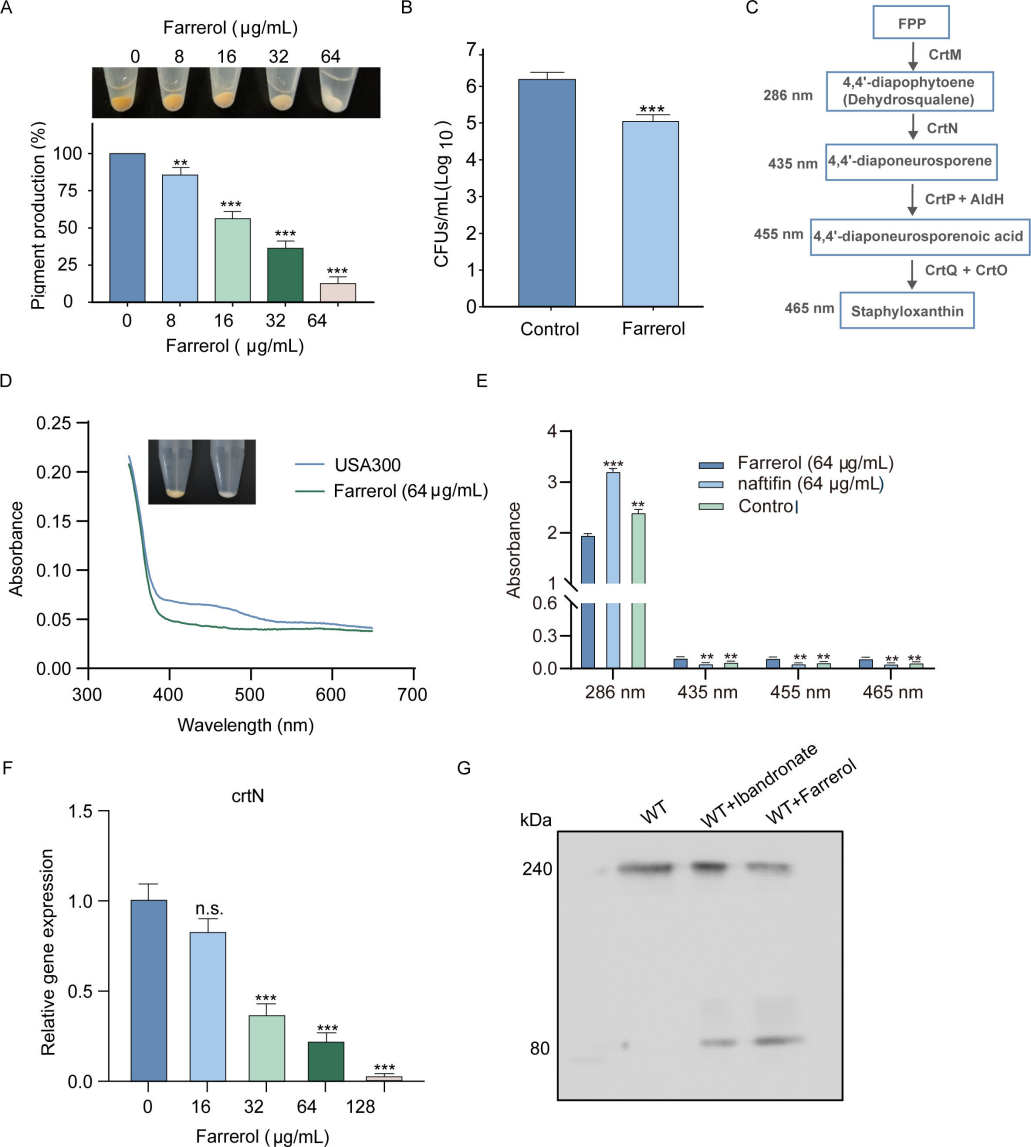
Impact of FA on the synthesis of pigments and oligomerization of PBP2a in *S. aureus* USA300. (**A**) Effect of FA on the deposition of pigmentation in *S. aureus* USA300. (**B**) The influence of FA on the sensitivity of *S. aureus* USA300 to H_2_O_2_-induced killing. (**C**) The biosynthesis process of *S. aureus* staphyloxanthin: from FPP to staphyloxanthin. (**D**) Full-spectrum absorbance analysis of the impact of FA on *S. aureus* pigment synthesis intermediates. (**E**) Effect of FA on the synthesis of *S. aureus* pigment intermediates, as evaluated by specific absorbance measurements at 286, 435, 455, and 465 nm. (**F**) Assessment of the perturbations in the expression of *crtN* in *S. aureus* USA300 under FA exposure. (**G**) Blue native PAGE and immunoblot analyses elucidating the state of PBP2a oligomerization in *S. aureus* USA300 in response to FA and ibandronate. n.s. represents no significant difference; ** represents *P* < 0.01; *** represents *P* < 0.001.

Given that FA does not affect the synthesis of WTA or the formation of septal bodies, we analyzed the effects of FA on FMMs, which are essential for PBP2a protein oligomerization. FMM proteins are composed of both staphyloxanthin and FloA. We found that a concentration of 64 µg/mL FA significantly inhibited pigment production by 43%. With an increased concentration of 256 µg/mL, pigment production was suppressed by 87% (Fig. 4A). Subsequent pigment extraction and hydrogen peroxide killing assays indicated that FA inhibits the synthesis of pigments in *S. aureus* in a dose-dependent manner, increasing susceptibility to oxidative killing (Fig. 4B).

Staphyloxanthin is synthesized through the staphyloxanthin biosynthetic pathway, which uses FPP as a substrate for further synthesis. Additionally, the maximum absorption peaks of the intermediate products varied (Fig. 4C). Methanol extraction of isoprenoid lipids followed by full-wavelength scanning revealed that the FA treatment group presented a reduction starting at approximately 350 nm, suggesting that FA may not affect FPP synthesis (Fig. 4D). Subsequent analysis at specific wavelengths for each intermediate product indicated that the levels of CrtM products did not decrease but rather increased, which is consistent with the effects of natifine, an inhibitor of the CrtN protein. This increase could be attributed to a compensatory mechanism. The products of *CrtN* were significantly reduced, indicating that FA impedes the function of *CrtN* (Fig. 4E). RT‒qPCR revealed a dose-dependent decrease in the transcription of the crtN gene, which is necessary for pigment synthesis, within the CrtOPQMN operon (Fig. 4F). These results suggest that FA reduces staphyloxanthin synthesis by directly decreasing *CrtN* expression.

According to the latest research, FMMs and PBP2a are linked. A deficiency in FMM components leads to the inability of PBP2a to form trimers on the membrane. We also tested whether FA could reduce PBP2a oligomerization. Ibandronate, which has been confirmed to interfere with PBP2a aggregation in *S. aureus*, served as a positive control. The results revealed that PBP2a can form trimers within bacteria after DSP crosslinking, and various degrees of oligomerization reduction occurred following treatment with the ispA inhibitor and FA (Fig. 4G).

In summary, these findings indicate that FA reduces staphyloxanthin synthesis by directly decreasing CrtN expression, further disrupting the composition of FMMs and ultimately leading to a decrease in the efficiency of PBP2a oligomerization.

### FA targets AgrA to disrupt the Agr quorum-sensing system

**Fig 5:**
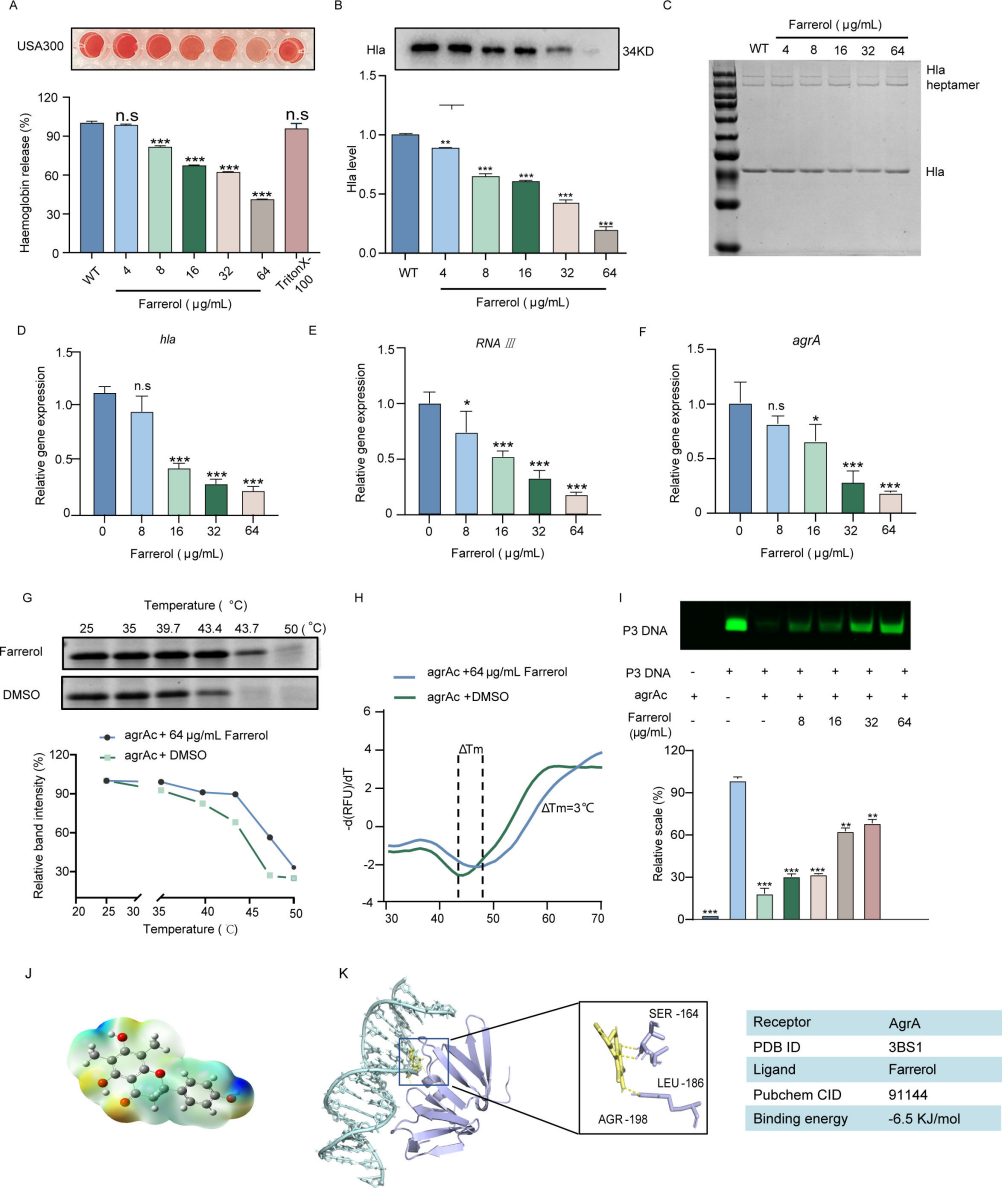
Disruption of the Agr system by FA through binding to the AgrAc protein. (**A**) Influence of FA on the hemolytic activity of *S. aureus* USA300. Triton was used as a positive control. (**B**) Immunoblot analysis examining the expression levels of Hla in the supernatant of *S. aureus* USA300 following treatment with various concentrations of FA. (**C**) Varying concentrations of FA did not affect Hla polymerization, as the quantity of Hla haptamer remained unchanged as the concentration of FA increased. (D‒F) RT‒qPCR analysis to investigate the effects of different concentrations of FA on the transcription levels of *hla*, *RNAIII*, and *agrA* in *S. aureus* USA300. (**G**) The thermal stability of AgrAc was influenced by FA, as determined by the CETSA method. (**H**) The TSA method was used to assess the effect of FA on the thermal stability of AgrAc. (**I**) The influence of various FA concentrations on the formation of AgAc-FAM-labeled oligonucleotide complexes was assessed through EMSA. (**J**) 3D diagram of ABEE molecular MEPs. (**K**) The molecular docking results revealed the critical amino acid residues involved in the binding of FA to AgrAc, suggesting that FA competes with AgrAc for DNA-binding sites. n.s. represents no significant difference; * represents *P* < 0.05; ** represents *P* < 0.01; *** represents *P* < 0.001.

Our investigation revealed that FA inhibits the hemolytic activity of MRSA USA300 in a dose-dependent manner. Remarkably, under the influence of a synergistic concentration of FA, the rate of inhibition of hemolytic activity by USA300 increased to 57.9% (Fig. 5A). The results obtained from western blot analyses revealed that FA suppressed the expression of hla in the supernatant of *S. aureus* USA300 (Fig. 5B). An oligomerization test revealed that FA did not affect the formation of heptamers from Hla (Fig. 5C). RT‒qPCR revealed a decrease in the transcription of Agr system-related genes, such as *hla*, *agrA*, and *RNAIII*, suggesting that the inhibitory effect of FA on hemolysis may be associated with the Agr quorum-sensing system (Fig. 5D through F). These compelling findings imply that the mechanism of action underlying the remarkable efficacy of FA may involve the modulation of hemolysin expression by directly targeting the Agr system of USA300 pathogens.

A prokaryotic expression vector for AgrAc was subsequently constructed, and the AgrAc protein was obtained. The degradation rate of the AgrAc protein was found to increase continually within the temperature range of 25–50°C via a CETSA. There was a noticeable difference in the proportion of degraded AgrAc protein in the DMSO and 64 µg/mL FA groups when the temperature increased to 43.7°C (Fig. 5G), providing preliminary evidence for an interaction between FA and the AgrAc protein. In the TSA, the degradation temperature (Tm) of the AgrAc protein was 47°C. Following treatment with 64 µg/mL FA, the protein degradation temperature increased to 50°C, with a Δ*Tm* >2°C, suggesting that FA can significantly affect the Tm of the AgrAc protein (Fig. 5H). According to the EMSA, the P3 promoter tagged with luciferase, a shorter piece of double-stranded DNA, was able to rapidly migrate according to gel electrophoresis. When the AgrAc protein bound to the P3 promoter, the migration speed of the P3 promoter significantly decreased, as indicated by only weak fluorescence. After treatment with 16–64 µg/mL FA, the fluorescence intensity at the P3 promoter gradually increased, indicating that FA inhibited the binding of the AgrAc protein to the P3 promoter and that the disappearance of fluorescence could be due to static quenching of the protein (Fig. 5I). Molecular docking was used to further analyze the binding site of FA on AgrAc. The conformational optimization of FA was performed via a Gaussian distribution, as shown in Fig. 5J. FA can bind to amino acids such as SER-164, LEU-186, and AGR-198 in the AgrA protein via hydrogen bonds, which can obstruct the binding of the AgrA protein to DNA. The docking score was calculated to be −7.0 kcal/mol (Fig. 5K). The results of the *in vitro* autophosphorylation assay for AgrC_C_ revealed that increasing concentrations of FA did not lead to a discernible decrease in the phosphorylation level of AgrC_C_ (Fig. S6). These findings indicate that FA does not affect the enzymatic activity of AgrC_C_.

Therefore, FA can bind to the AgrAc protein, thereby inhibiting the binding of AgrA to the P3 promoter. In summary, these results further substantiate our hypothesis that FA directly interacts with AgrA, which regulates the expression of virulence factors such as Hla by inhibiting the Agr system, thereby weakening the pathogenicity of *S. aureus* to achieve a synergistic antimicrobial effect.

### Protective effects of FA combined with cefepime in mice with *S. aureus*-induced pneumonia

**Fig 6:**
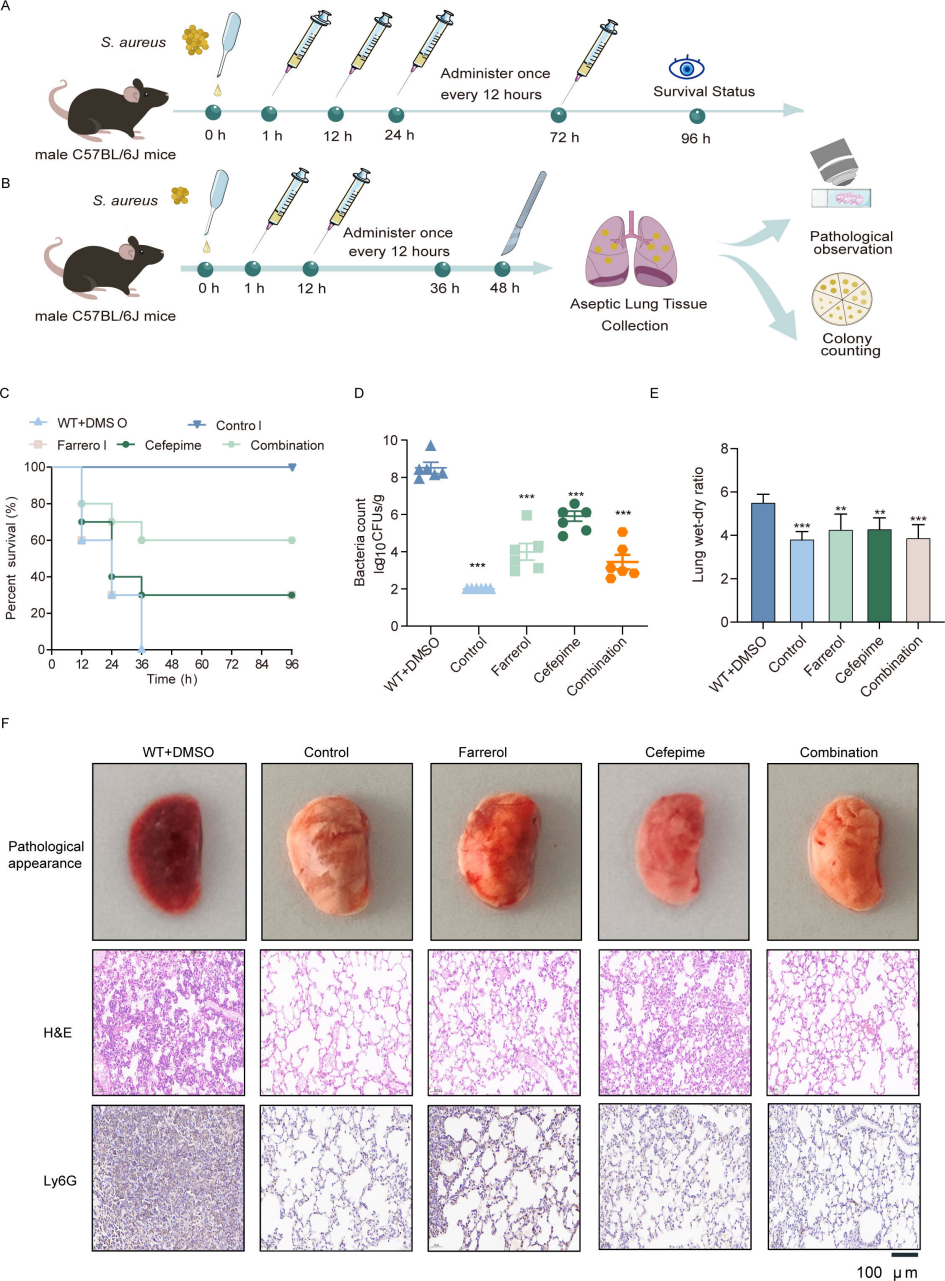
FA and cefepime combination therapy for mice with pneumonia induced by *S. aureus* USA300. (**A**) A pneumonia model was established via the nasal delivery of *S. aureus*, and treatments were given 1 h after infection and every 12 h thereafter to assess survival. (**B**) Wild-type (WT) refers to the *S. aureus* USA300 infection group. Sublethal doses of *S. aureus* were nasally administered, followed by treatment at 1 h and every 12 h post-infection, and pathology and colony counts were evaluated at 48 h. (**C**) Comparison of 96-h survival rates among mice left untreated or treated with DMSO, FA, cefepime, or a combination of FA and cefepime. (**D**) Quantification of the bacterial load in the lung tissues across different treatment groups. (**E**) Analysis of the lung wet‒dry weight ratios in different treatment groups of mice. (**F**) Investigation of pathological and histopathological changes in lung tissues across all groups following *S. aureus* infection via hematoxylin and eosin (H&E) and LY6G immunohistochemical analysis. ** represents *P* < 0.01. *** represents *P* < 0.001.

To evaluate the *in vivo* effects of FA and cefepime against *S. aureus*, we initially established a murine model of *S. aureus*-induced pneumonia. Lethal *S. aureus* USA300 (2 × 10^8^ CFU) was intranasally inoculated into 7-week-old mice from each group. Two hours after infection, 50 mg/kg FA or 50 mg/kg cefepime was intraperitoneally injected into the mice. The treatment was repeated every 12 h, and the mortality rate of the infected animals was assessed after 96 h (Fig. 6A). After 48 h, the wild-type (WT) group, representing the *S. aureus* USA300 infection-only group, exhibited complete mortality. Treatment with either FA or cefepime alone increased survival to 30% compared to the infection group, whereas the combination of FA and cefepime resulted in a 60% survival rate. These findings suggest that the combination of FA and cefepime significantly enhances survival in mice with acute pneumonia, particularly during the early stages of infection (Fig. 6C). At 48 h post*-S. aureus* infection, the lung tissues were homogenized and plated for bacterial enumeration. As shown in Fig. 6B and D, the lungs of the WT group mice were laden with a high quantity of bacteria (8.68 ± 0.70 CFU/g). Compared with those in the WT group, the bacterial loads in the lung tissue of the FA and cefepime groups were notably lower (5.54 ± 0.65 and 6.34 ± 0.73 log10 CFU/g, respectively). FA reduced the lung W/D weight ratio, suggesting that FA reduces pulmonary edema (Fig. 6E).

Next, we analyzed the pathological alterations in mice with pneumonia induced by *S. aureus* USA300 following combination therapy. As shown in Fig. 6F, the lung tissue of the mice in the combination group was pinkish and spongy and exhibited less localized infection than the untreated mouse lung tissue, which was dark red, indicating severe infection. Subsequent H&E staining and immunocytochemistry for Ly6G analysis revealed that the majority of alveolar spaces in the lung tissue of the *S. aureus* USA300-infected group were infiltrated by inflammatory cells and neutrophils, which was noticeably mitigated following individual treatment with FA or cefepime. In conclusion, the coadministration of FA and cefepime notably improved survival rates in mice, curtailed *S. aureus* colonization in lung tissue, ameliorated tissue damage, and effectively interrupted the progression of infection in a mouse pneumonia model.

## DISCUSSION

In recent years, interest in antibacterial infections through large-scale compound screening strategies has increased. However, several traditional virulence-targeting strategies are associated with resistance. Given the intricate network of regulatory mechanisms within bacteria, these targets often play multifaceted roles. Consequently, many targets, such as WTA, Agr, and pigments pertaining to FMMs, have been revealed to possess dual capabilities, impacting both resistance and virulence.

WTA is a ribitol phosphate polymer that covalently links with PG and plays an important role in the synthesis of the cell wall of *S. aureus*, participating in maintaining cell shape and host colonization (53, 54). The biosynthesis pathway of WTA can be divided into early and late stages. Owing to its intrinsic properties, the suppression of genes early in the WTA biosynthesis pathway does not lead to cell death, whereas the inhibition of genes late in the pathway does. This could be attributed to the generation of toxic intermediates or the usurpation of UPPs interfering with cell wall synthesis (55). Drugs that inhibit WTA synthesis can interfere with the localization of PBP4 and the stability of PBP2a, thereby restoring the sensitivity of MRSA to β-lactam antibiotics (56). Furthermore, WTA is also regulated by the Agr system. In *S. aureus* with a deficient agr system, shorter K-WTA is produced, reducing the extracellular density of WTA and increasing adhesiveness. In contrast, in *S. aureus*, which has a complete Agr system, the agr system suppresses the expression of TarK, leading to the expression of longer L-WTA, which reduces the adhesiveness of *S. aureus* and increases the extracellular density of WTA (55). These effects were initially misleading, as they led us to hypothesize that FA exerts its synergistic effects by interfering with WTA synthesis. The observed decrease in WTA density under TEM was due to Agr inhibition.

FMMs in *S. aureus*, which are composed of flotillin proteins and staphyloxanthin, are lipid raft structures. FMMs assist the type VII secretion system in secretion and signal transmission, promoting the efficient oligomerization of extracellular protein complexes (57). Although FMMs are not essential for the extracellular survival of *S. aureus*, they increase the oligomerization and stability of PBP2 and PBP2a, thereby increasing the antibiotic resistance of MRSA (17). In mutants deficient in staphyloxanthin biosynthesis, the total number of FMMs decreases, and the probability of the remaining FMMs being distributed in the septum further decreases. To interfere with the function of FMMs, researchers have focused on their constituents, staphyloxanthin and the FloA protein. Staphyloxanthin inhibitors have been widely studied, but few of these agents exhibit satisfactory pharmacokinetics. Staphyloxanthin is a triterpenoid carotenoid that usually acts as a reductant and antioxidant in *S. aureus* (58). Its synthesis pathway is composed of the Crt*OPQMN* operon, which initially synthesizes pigment precursors via the nonmevalonate pathway, followed by the staphyloxanthin pathway (38). Moreover, staphyloxanthin assists *S. aureus* in evading host immunity (36) and combatting reactive oxygen species (59). Clinically, numerous drugs can inhibit the synthesis of pigments in *S. aureus*. Statins are a particularly promising choice. Many clinical and epidemiological studies have shown that patients with severe infections (including sepsis, pneumonia, and bacteremia) who are treated with statins demonstrate significant improvements (16). Although the target of FA in the inhibition of pigments is unclear, its excellent *in vivo* pharmacological properties make it a potential adjuvant for treating *S. aureus* pneumonia. FA also inhibits pigment synthesis. In our experiments, FA was found to reduce the transcription level of *crtN*, thereby decreasing the number of FMMs in *S. aureus*. FA enhances the sensitivity of *S. aureus* to β-lactams and inhibits MRSA resistance to antibiotics by reducing PBP2a protein oligomerization and inhibiting the function of BlaZ. The inhibitory effect of FA on *S. aureus* pigments also prevents *S. aureus* from escaping intracellular ROS, as does the ROS response induced by β-lactam antibiotics (60).

In *S. aureus*, the upregulation of virulence factors by the Agr system is necessary in acute pneumonia infection animal models (61), likely because the virulence factors are regulated and secreted by the Agr system. Oxacillin has the ability to attenuate MRSA virulence. This effect is predicated on the capacity of oxacillin to inhibit the MRSA Agr system and alter the cell wall structure, indirectly increasing the sensitivity of MRSA to host-derived killing (62). An effective reduction in MRSA virulence and resistance can be achieved through the combination of Agr inhibitors with β-lactam antibiotics. In previous investigations, FA was demonstrated to modulate the expression of antimicrobial peptides, thereby diminishing the internalization of *S. aureus* in bovine mammary epithelial cells (63). Consistent with these findings, our results also demonstrate that FA effectively inhibits the production of *S. aureus* Hla, a major virulence factor involved in immune evasion and tissue damage. This inhibition occurs through the suppression of the Agr system, aligning with previous literature, which also reported FA’s ability to inhibit Hla production (64). In addition, FA has a broad spectrum of pharmacological effects, including antioxidative, anti-inflammatory, vasodilatory, antitumorigenic, and antibacterial effects (65). In this study, we systematically investigated the impacts of FA on the resistance and virulence of *S. aureus*, providing insights into the role of natural compounds in targeting novel mechanisms of MRSA infection.

Cefepime, a fourth-generation cephalosporin, is primarily designed to target gram-negative pathogens and exhibits limited intrinsic activity against MRSA due to its poor affinity for PBP2a, which is encoded by the *mecA* gene and confers β-lactam resistance in MRSA (66). This inherent limitation underscores the challenges of using cefepime as a monotherapy against MRSA infections. Cefepime exerts its antibacterial activity by targeting conventional PBPs, including PBP1, PBP2, and PBP3, which are essential for bacterial cell wall synthesis in MSSA. However, in MRSA, the presence of PBP2a with low affinity for β-lactams significantly diminishes cefepime’s efficacy. Additionally, MRSA’s production of β-lactamases further degrades cefepime, compounding its limitations in treating MRSA infections. Nevertheless, recent research has increasingly focused on strategies to enhance the efficacy of β-lactam antibiotics by targeting resistance and virulence pathways.

In this context, our study highlights FA as a novel adjuvant capable of re-sensitizing MRSA to cefepime through a dual mechanism: inhibition of β-lactamase activity and attenuation of virulence factors such as pigment production, pore-forming toxins, and the Agr system. By weakening bacterial defenses and reducing pathogenicity, FA creates a favorable environment for cefepime to exert antimicrobial effects via synergistic interactions. This approach not only restores the antimicrobial potential of cefepime but also complements traditional bactericidal strategies with virulence attenuation, representing a paradigm shift in combating antibiotic resistance.

## Data Availability

The data supporting the findings of this study are available in the paper and its supplemental material.

## SUPPLEMENTAL MATERIAL

Supplementary Material aac.01551-24-s0001.docx Fig. S1 to S6; Tables S1 to S4.

ASM does not own the copyrights to Supplemental Material that may be linked to, or accessed through, an article. The authors have granted ASM a non-exclusive, world-wide license to publish the Supplemental Material files. Please contact the corresponding author directly for reuse.
