# Supplementary material for: Mechanistic insights into the multitarget synergistic efficacy of farrerol and β-lactam antibiotics in combating methicillin-resistant Staphylococcus aureus
Source: Antimicrob Agents Chemother. 2025 Feb 28;69(4):e01551-24. doi: 10.1128/aac.01551-24 (PMC11963547; doi:10.1128/aac.01551-24)
Supplement: Supplementary Material — Fig. S1 to S6; Tables S1 to S4. [file aac.01551-24-s0001.docx]

**Supplementary Information**

**Mechanistic Insights into the Multitarget Synergistic Efficacy of Farrerol and β-Lactam Antibiotics in Combating Methicillin-Resistant *Staphylococcus aureus***

Hangqian Yu^1,2#^, Li Wang^3#^, Xin Liu^1^, Jianze Zheng^1^, Hua Xiang^4^, Yanyang Zheng^4^, Dongmei Lv^1^, Jingjing Liu^1^, Yuxin Zhang^1^, Jiazhang Qiu^2*^, Dacheng Wang^1*^

^1^ College of Animal Science, Jilin University, Changchun, China

^2^ State Key Laboratory for Diagnosis and Treatment of Severe Zoonotic Infectious Diseases, Key Laboratory for Zoonosis Research of the Ministry of Education, College of Veterinary Medicine, Jilin University, Changchun, China

^3^ Clinical Medical College, Changchun University of Chinese Medicine, Changchun, China

^4^ College of Animal Medicine, Jilin Agricultural University, Changchun, China

^#^ These authors contributed equally to this work.

*Corresponding author: Dacheng Wang, Email: wangdc@jlu.edu.cn; Jiazhang Qiu, Email: qiujz@jlu.edu.cn

**Table of contents**

Figure S1. Quality test report of farrerol (FA).

Figure S2. Time‒kill kinetics of the MRSA strain USA300 exposed to varying concentrations of cefepime and FA in combination.

Figure S3. Protective effects of FA on A549 cells infected with MRSA USA300.

Figure S4 Safety assessment of FA.

Figure S5 Penicillin G degradation analysis and β-lactamase activity determination.

Figure S6 Effect of FA on the autophosphorylation of AgrCc.

Table S1. Strains used in this study.

Table S2. Primers used in this study.

Table S3. Effect of FA in combination with β-lactams against MRSA USA300.

Table S4. Effect of FA in combination with β-lactams against MSSA 29213.

**Figure S1. Quality test report of farrerol (FA)**

**
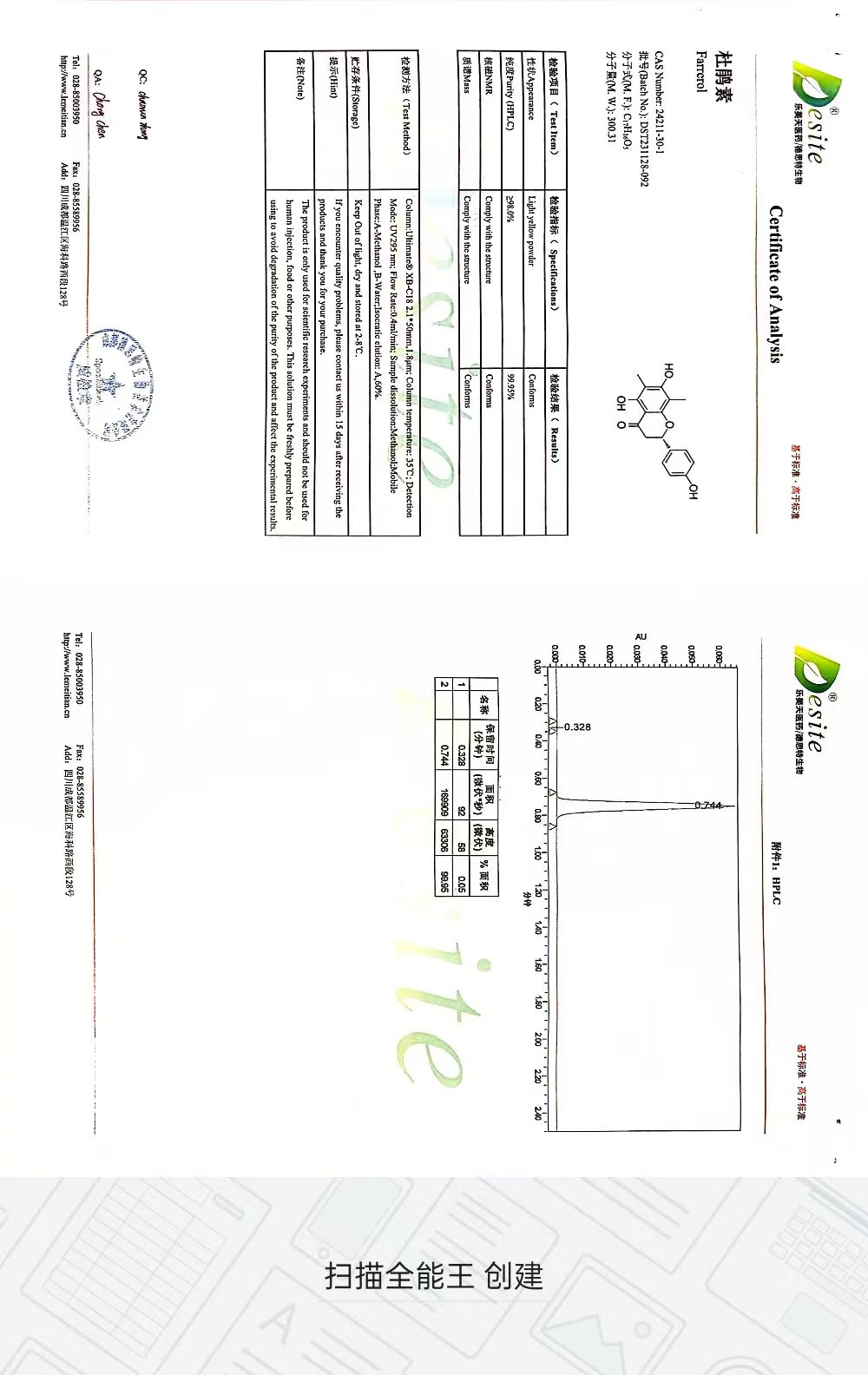
**

Figure S1. Quality test report of farrerol. HPLC chromatogram of farrerol. The purity of the farrerol was 99.95%.

**Figure S2. Time‒kill kinetics of the MRSA strain USA300 exposed to varying concentrations of cefepime and FA in combination**

**
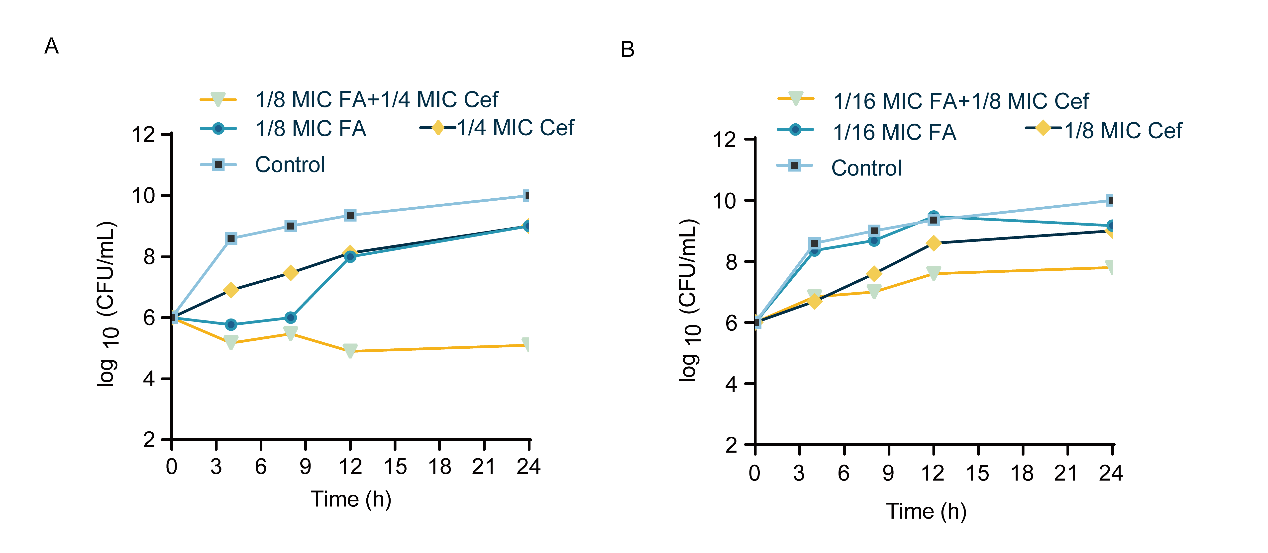
**

Figure S2. Time‒kill kinetics of the MRSA strain USA300 exposed to varying concentrations of cefepime and FA in combination. A represents the 1/4 MIC of cefepime and the 1/8 MIC of FA, whereas B denotes the 1/8 MIC of cefepime coupled with the 1/16 MIC of FA.

**Figure S3. Protective effects of FA on A549 cells infected with MRSA USA300.**

**
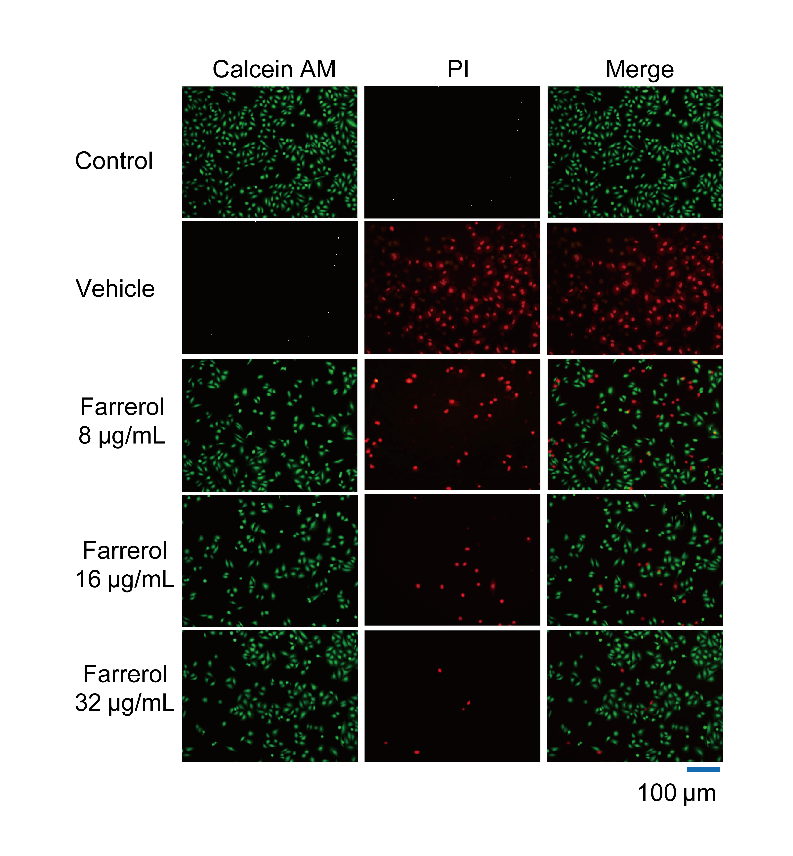
**

Figure S3. Protective effects of FA on A549 cells infected with MRSA USA300, as determined by imaging cells stained with live/dead reagents and visualized under a fluorescence microscope. Green fluorescence signifies live cells, whereas red fluorescence marks dead cells.

**Figure S4 Safety assessment of FA.**


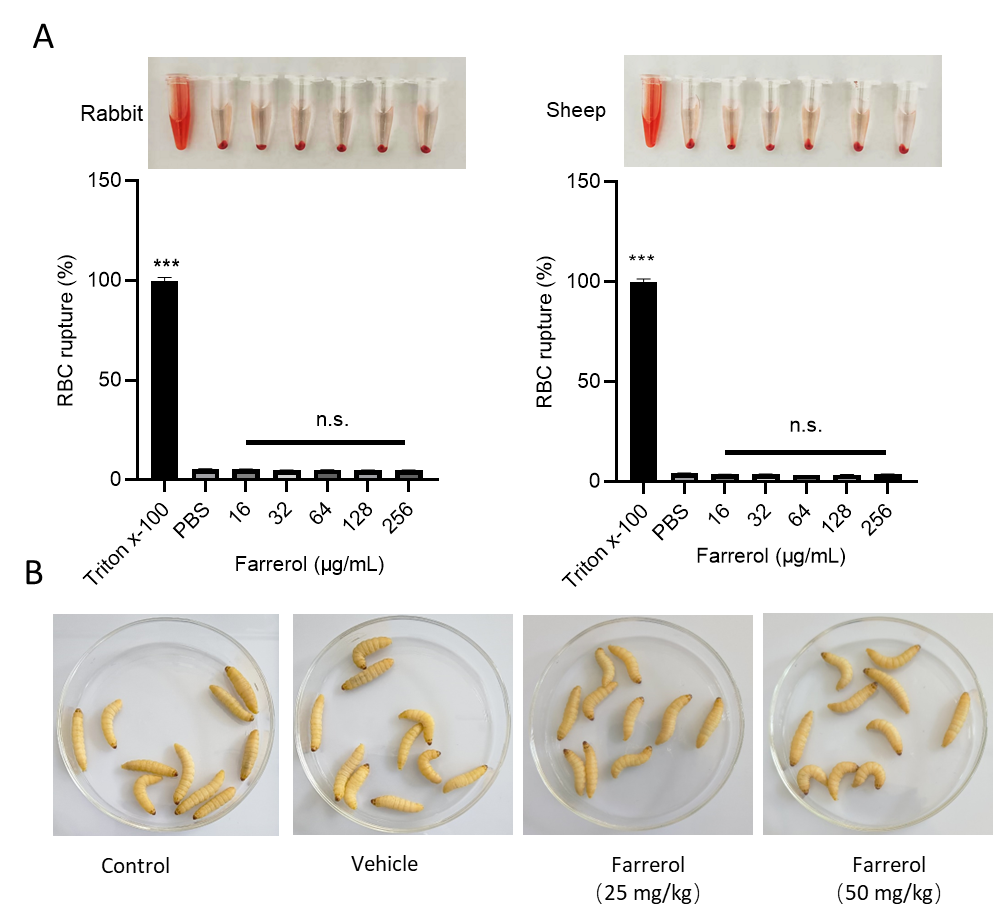


Figure S4 Safety assessment of FA. A. Analysis of the effects of FA on red blood cells across various concentrations. B. Investigating the effects of FA on *Galleria mellonella* Larvae Well-being. This figure presents the results from administering doses of 25 mg/kg and 50 mg/kg FA, with a focus on its impact on the melanization process and the survival rates of *Galleria mellonella* larvae. The data indicate that, within these dosage parameters, no melanization or fatalities were observed.

**Figure S5. Penicillin G degradation analysis and β-lactamase activity determination**


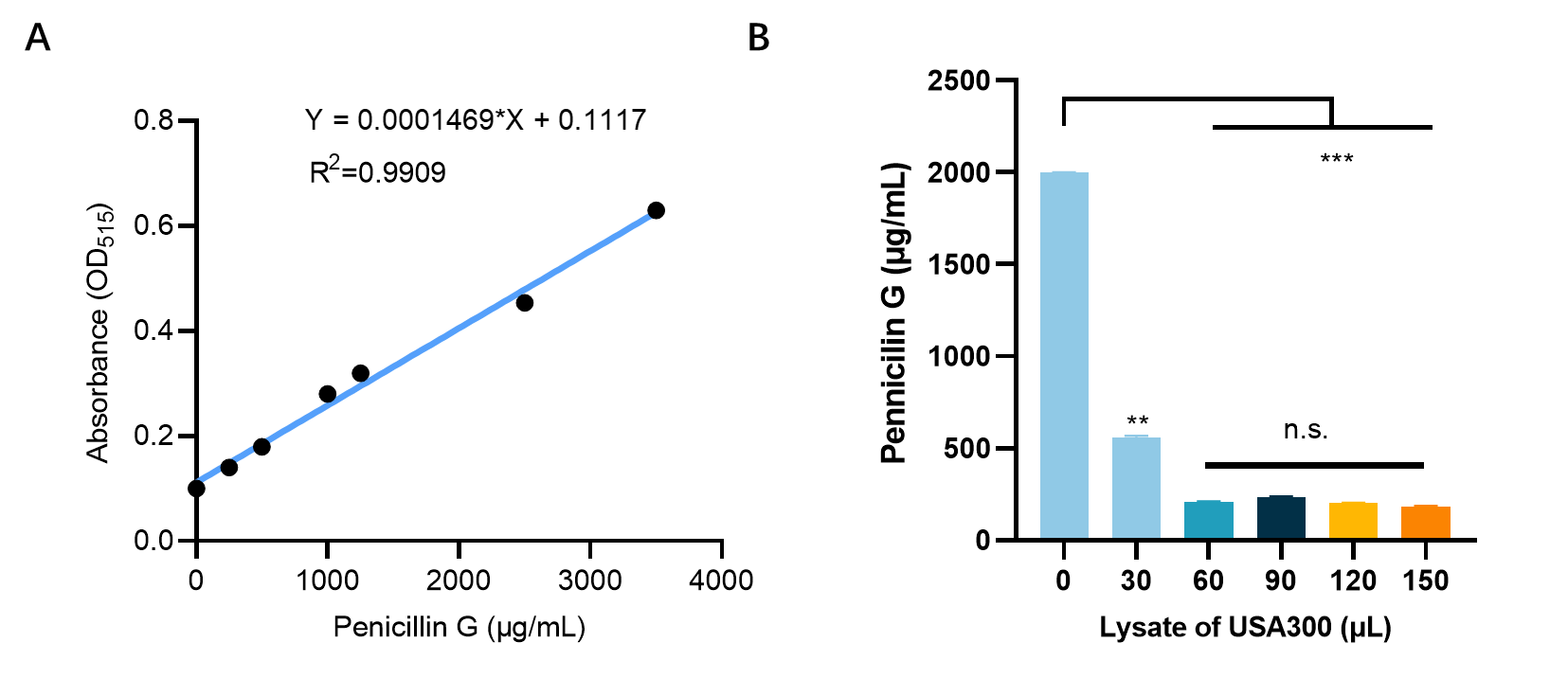


**Figure S5. Penicillin G degradation analysis and β-lactamase activity determination.**

(A) Standard curve for penicillin G determination via the hydroxylamine assay. Penicillin G was dissolved in sodium citrate and HCl buffer, with varying volumes added to achieve a final volume of 1 mL. The OD515 was measured after the addition of neutral light amine, ethanol, and ferric ammonium sulfate, confirming a linear relationship between the penicillin G concentration and the OD_515_ value. (B) Relationship between the β-lactamase concentration and penicillin G degradation. Various concentrations of USA300 lysate were reacted with penicillin G (final concentration 2000 μg/mL) for 30 minutes. The results were used to establish a standard curve correlating the β-lactamase concentration with the extent of penicillin G degradation.

**Figure S6 Effect of FA on the autophosphorylation of AgrCc.**


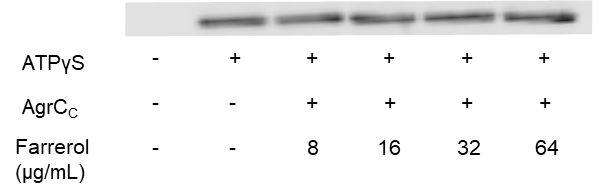


Figure S6 Effect of FA on the autophosphorylation of AgrCc. With increasing FA concentration, there was no decrease in the phosphorylation of AgrCc, indicating that FA does not impinge upon the autophosphorylation process intrinsic to AgrCc kinase activity.

**Table S1. Strains used in this study**

| Strains | Source |
| --- | --- |
| MRSA | ATCC^®^ BAA-1717™ (USA300-HOU-MR) |
| MSSA | ATCC 29213 |
| pET28a::*blaZ*(DE_3_) | This study |
| pET28a::*agrAc*(DE_3_) | This study |
| pET28a::*agrCc*(DE_3_) | This study |
| pET28a::Hla (DE_3_) | This study |

ATCC, American Type Culture Collection; MRSA, methicillin-resistant *Staphylococcus aureus*

MSSA, methicillin-sensitive *Staphylococcus aureus*

**Table S2 Primers used in the study**

| Primers | Sequence (5′→ 3′) | Purpose |
| --- | --- | --- |
| *blaTEM-1-*F | TCCGCTCATGAGACAATAACC | PCR |
| *blaTEM-1-*R | TTGGCTGACAGTTACCAATG | PCR |
| *blaCTX-M*-F | TCTTCCAGAATAAGGAATCCC | PCR |
| *blaCTX-M*-R | CCGTTTCCGCTATTACAAAC | PCR |
| *blaSHV-*F | TGGTTATGCGTTATATTCGCC | PCR |
| *blaSHV-*R | GGTTAGCGTTGCCAGTGCT | PCR |
| *blaOXA-2-*F | AAGAAACGCTACTCGCCTGC | PCR |
| *blaOXA-2-*R | CCACTCAACCCATCCTACCC | PCR |
| *blaOXA-10-*F | GTCTTTCGAGTACGGCATTA | PCR |
| *blaOXA-10-*R | ATTTTCTTAGCGGCAACTTAC | PCR |
| *blaVEB*-F | GATAGGAGTACAGACATATG | PCR |
| *blaVEB*-R | TTTATTCAAATAGTAATTCCACG | PCR |
| *blaPER*-F | ATGAATGTCATCACAAAATG | PCR |
| *blaPER*-R | TCAATCCGGACTCACT | PCR |
| *blaGES-F* | ATGCGCTTCATTCACGCAC | PCR |
| *blaGES-R* | CTATTTGTCCGTGCTCAGG | PCR |
| *blaZ-F* | CGCGGATCCAAAGAGTTAAATGATTTA | PCR |
| *blaZ-R* | CCGCTCGAGTCAAAATTCCTTCTATACACT | PCR |
| *16S*-F | TGATCCTGGCTCAGGATGA | qRT‒PCR |
| *16S*-R | TTCGCTCGACTTGCATGTA | qRT‒PCR |
| *hla*-F | GGTATATGGCAATCAACTT | qRT‒PCR |
| *hla-R* | CTCGTTCGTATATTACATCTAT | qRT‒PCR |
| *RNAIII*-F | AATTAGCAAGTGAGTAACATTTGCTAGT | qRT‒PCR |
| *RNAIII*-R | GATGTTGTTTACGATAGCTTACATGC | qRT‒PCR |
| *agrA*-F | GCAGTAATTCAGTGTATGTTCA | qRT‒PCR |
| *agrA*-R | TATGGCGATTGACGACAA | qRT‒PCR |
| *agrA_c_*-F | CGCGGATCCATGGATAATAGCGTTGAAACG | PCR |
| *agrA_c_*-R | CCGCTCGAGCGGTATTTTTTTAACGTTTCTCAC | PCR |
| *P3*-F | AATTTTTCTTAACTAGTCGTTTTTTATTCTTAACTGTAA | EMSA |
| *P3*-R | TTACAGTTAAGAATAAAAAACGACTAGTTAAGAAAAATT | EMSA |
| *agrC_c_*-F | CTGGGATCCAAAGAGATGAAATATAAACG | PCR |
| *agrC_c_*-R | CTGGTCGACCTAGTTGTTAATAATTTCAAC | PCR |
| *crtN*-F | CAGTGATTGGTGCAGGTGTC | qPCR |
| *crtN*-R | CATACGCCCGCCTACATTAT | qPCR |
| *hla*-clone-F | CGCGGATCCGCAGATTCTGATATTAATATTAAAAC | PCR |
| *hla-* clone-R | CCGCTCGAGTTAATTTGTCATTTCTTCTTTTTC | PCR |

**Table S3. Effect of FA in combination with β-lactams on MRSA USA300**

| Antibiotic | MIC _Antibiotic_(μg/mL) | FIC Antibiotic | MIC farrerol(μg/mL) | FIC farrerol | FICI |
| --- | --- | --- | --- | --- | --- |
| Cefoxitin | 16 | 0.25 | 512 | 0.125 | 0.375 |
| Cefepime | 32 | 0.25 | 512 | 0.125 | 0.375 |
| Ceftriaxone | 32 | 1 | 512 | 0.125 | 1.125 |
| Ceftazidime | 64 | 1 | 512 | 0.125 | 1.125 |
| Cefotaxime | 32 | 0.5 | 512 | 0.125 | 0.625 |
| Ampicillin | 512 | 0.125 | 512 | 0.125 | 0.250 |
| Penicillin G | 1024 | 0.125 | 512 | 0.125 | 0.250 |

**Table S4.** **Effect of FA in combination with β-lactams against MSSA 29213**

| Antibiotic | MIC Antibiotic(μg/mL) | FIC Antibiotic | MIC farrerol(μg/mL) | FIC farrerol | FICI |
| --- | --- | --- | --- | --- | --- |
| Cefoxitin | 1 | 1 | 512 | 0.125 | 1.125 |
| Cefepime | 1 | 1 | 512 | 0.125 | 1.125 |
| Ceftriaxone | 1 | 1 | 512 | 0.125 | 1.125 |
| Ceftazidime | 4 | 1 | 512 | 0.125 | 1.125 |
| Cefotaxime | 1 | 1 | 512 | 0.125 | 1.125 |
| Ampicillin | 8 | 0.25 | 512 | 0.125 | 0.375 |
| Penicillin G | 32 | 0.125 | 512 | 0.125 | 0.250 |
